# Mechanism of glutathionylation of the active site thiols of peroxiredoxin 2

**DOI:** 10.1016/j.jbc.2025.108503

**Published:** 2025-04-11

**Authors:** Alexander V. Peskin, Flavia C. Meotti, Nicholas J. Magon, Luiz F. de Souza, Armindo Salvador, Christine C. Winterbourn

**Affiliations:** 1 Mātai Hāora - Centre for Redox Biology and Medicine, Department of Pathology and Biomedical Science, University of Otago, Christchurch, New Zealand; 2 Department of Biochemistry, Chemistry Institute, University of Sao Paulo, Sao Paulo, Sao Paulo, Brazil; 3 CNC-UC - Centre for Neuroscience Cell Biology, University of Coimbra, Coimbra, Portugal; 4 CiBB - Centre for Innovative Biomedicine and Biotechnology, University of Coimbra, Coimbra, Portugal; 5 Coimbra Chemistry Center - Institute of Molecular Sciences (CQC-IMS), University of Coimbra, Coimbra, Portugal; 6 Institute for Interdisciplinary Research, University of Coimbra, Coimbra, Portugal

**Keywords:** peroxiredoxin, glutathionylation, thiol, kinetic modeling, antioxidant, redox signaling

## Abstract

Peroxiredoxin 2 (Prdx2) undergoes ready glutathionylation, and glutaredoxin-catalyzed deglutathionylation provides an alternative mechanism to thioredoxin/thioredoxin reductase for recycling the reduced protein (Peskin *et al.* JBC 216, 3053, 2016). To elucidate the mechanism of glutathionylation, we have carried out kinetic studies using stopped flow and SDS PAGE plus product analysis by mass spectrometry. Kinetic modeling shows a mechanism in which exchange of Prdx2 disulfide with physiological concentrations of GSH occurs over seconds to minutes, initially at one active site to produce glutathionylated dimers linked by one disulfide. Exchange with GSH yields glutathionylation at both the peroxidatic (C_*P*_) and resolving cysteines (C_*R*_), the former predominating. Rate constants of 1.5 M^−1^s^−1^ and 0.021 s^−1^ were determined for exchange-mediated glutathionylation and deglutathionylation. Similar exchange reactions subsequently occur at the second active site. The rate of reaction of the C_*P*_ sulfenic acid of WT Prdx2 with GSH (k = 10 M^−1^s^−1^) is 8 to 30 fold slower than when C_*R*_ is mutated to Ser, Trp, or Asp and this reaction cannot effectively compete with intramolecular condensation. Consequently, when H_2_O_2_ reacts with reduced Prdx2 in the presence of GSH, the initial product is predominately the Prdx disulfide and glutathionylation subsequently occurs by exchange. However, glutathionylation of C_*R*_ in the presence of H_2_O_2_ facilitates condensation of C_*P*_ sulfenic acid with GSH to give diglutathionylated products and suppresses hyperoxidation. This displaces equilibria and accelerates the conversion of Prdx2 to monomeric species. These results have implications for understanding the mechanism of relays between Prdx2 and other thiol proteins.

Peroxiredoxin 2 (Prdx2) is a ubiquitously expressed mammalian cytoplasmic 2-Cys Prdx that reacts extremely rapidly with H_2_O_2_ and other peroxides. Along with other Prdxs, it plays a major role in antioxidant defense and the regulation of peroxide homeostasis (1, 2). Prdxs also participate in redox signaling by acting as sensors of peroxides and transmitting oxidizing equivalents to other thiol proteins *via* a relay mechanism (3, 4). The catalytic unit of Prdx2 is a homodimer that is noncovalently associated in its reduced state and disulfide-linked when oxidized. Each monomer contains an active site Cys (C52; designated C_*P*_) which undergoes oxidation to a sulfenic acid and condensation with the resolving Cys (C172; designated C_*R*_) on the opposing chain to form a disulfide. The dimeric unit therefore contains two active sites that undergo sequential oxidation to form one then two interchain disulfide bonds. Reduction of the disulfides completes the catalytic cycle of peroxide removal and enables Prdx2 to function as a regulator of peroxide activity or antioxidant. Recycling of Prdx2 is most readily accomplished by reduced thioredoxin (Trx), which reduces the disulfide and is regenerated by Trx reductase plus NADPH.

Prdx2 in the presence of GSH undergoes glutathionylation of its active site thiols (5). These mixed disulfides are readily reduced by glutaredoxin (Grx)/GSH, and this system provides an alternative to the Trx system for regenerating the reduced Prdx. Glutathionylation is also significant as a potential mechanism for altering the properties of Prdx2, such as its peroxide sensitivity, oligomeric state, and protein–protein interactions (6). In addition, formation of a mixed disulfide with GSH can be considered as a model for mixed disulfide formation between Prdx2 and other thiol proteins, which occurs in redox relays. Although we know from previous studies that oxidation of Prdx2 in the presence of GSH results in the formation of mixed disulfides, it has not been established whether this occurs by condensation of GSH with Prdx2-SOH (Reaction 1), by thiol-exchange between GSH and Prdx2 disulfide (reaction 2), or by a combination of both mechanisms.

(1) PrdxSOH+GSH→PrdxS-SG+H2O

(2) PrdxS-SPrdx+GSH⇌PrdxS-SG+PrdxSH

To elucidate the mechanism, we have performed kinetic studies using stopped flow and SDS-PAGE plus product analysis by mass spectrometry (MS) and carried out modeling analysis to obtain rate and equilibrium constants to fit the data. Our findings show that condensation of Prdx2 sulfenic acid with GSH is slow and glutathionylation occurs initially by an exchange equilibrium mechanism. However, glutathionylation of C_*R*_ facilitates the reaction of C_*P*_ -sulfenic acid with GSH, and a combination of GSH and H_2_O_2_ gives extensive formation of mono- and di-glutathionylated products.

## Results

### Terminology

**Figure 1:**
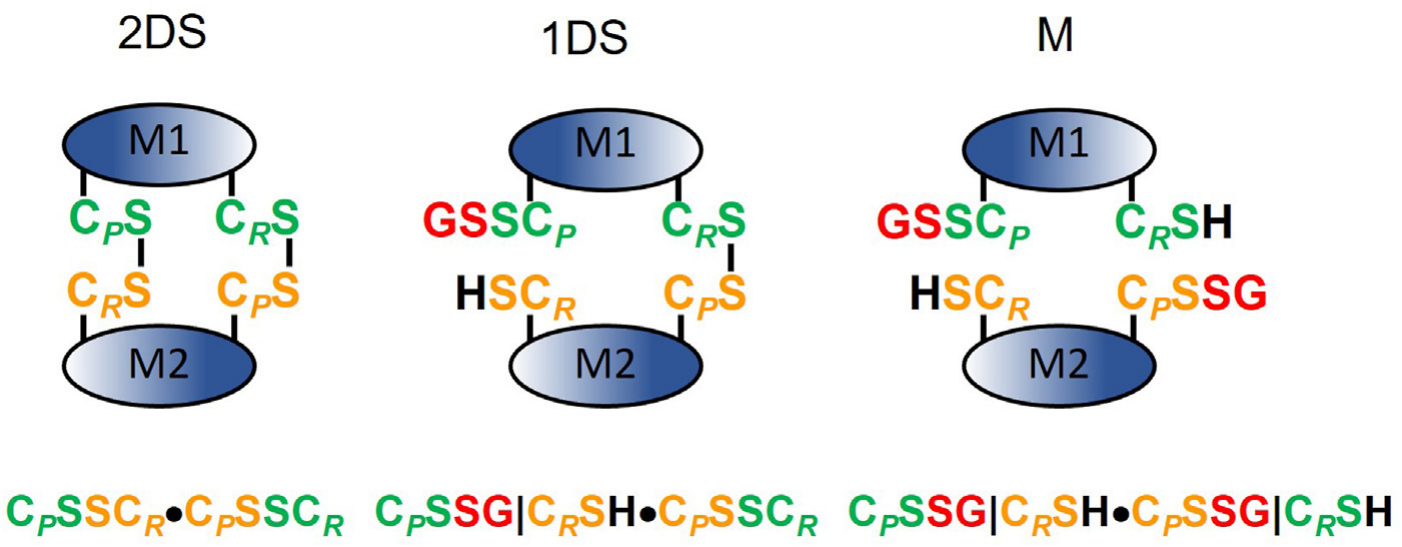
**Convention used to represent the thiol redox state of Prdx2 in the 2 disulfide, 1 disulfide, and monomer (M) state in its reaction with GSH.** A dimeric unit with its two active sites is shown with the peroxidatic and resolving cysteines designated C_*P*_ and C_*R*_. Illustrated here are monoglutathionylated forms with the glutathione conjugated to C_*P*_. The state of each active site is represented to each side of the “•” symbol, and the state of each Cys side group in an active site is separated by a “|” symbol unless the site is in disulfide form. The cysteines from the different subunits are color coded *green* or *yellow*. A similar convention is used where there is glutathionylation of C_*R*_ or diglutathionylation. When the focus is on the state of one of the sites and the state of the other site is considered invariant, the latter is omitted for simplicity. Note that the reverse of glutathionylation in which C_*P*_-SH or C_*R*_-SH attacks the mixed disulfide at, respectively, C_*R*_ or C_*P*_ to form a C_*P*_-C_*R*_ disulfide and release GSH, we will denote as “self-deglutathionylation.”

The results we present have been obtained using SDS-PAGE and protein mass spectrometric methods that break noncovalent associations. Therefore, we have used monomer/dimer terminology to refer to noncovalently associated and disulfide-linked forms of Prdx2. Thus, monomer refers to forms containing no interchain disulfide, and dimer refers to oxidized forms containing one or two disulfides. This terminology is distinct from that used for considering quaternary structure of 2-Cys Prdxs, where dimer refers to the functional homodimeric unit, which associates noncovalently and can exist in both reduced and disulfide-linked forms and which undergoes further reversible association to form noncovalent decamers. We will designate such a functional unit by “dimeric unit.” A schematic of the conventions used for designating the different Prdx2 forms is shown in Figure 1. The peroxidatic and resolving thiols and sulfenic acids are written in protonated form in symbols and abbreviations for simplicity.

### Condensation of GSH with Prdx2 sulfenic acid

**Figure 2:**
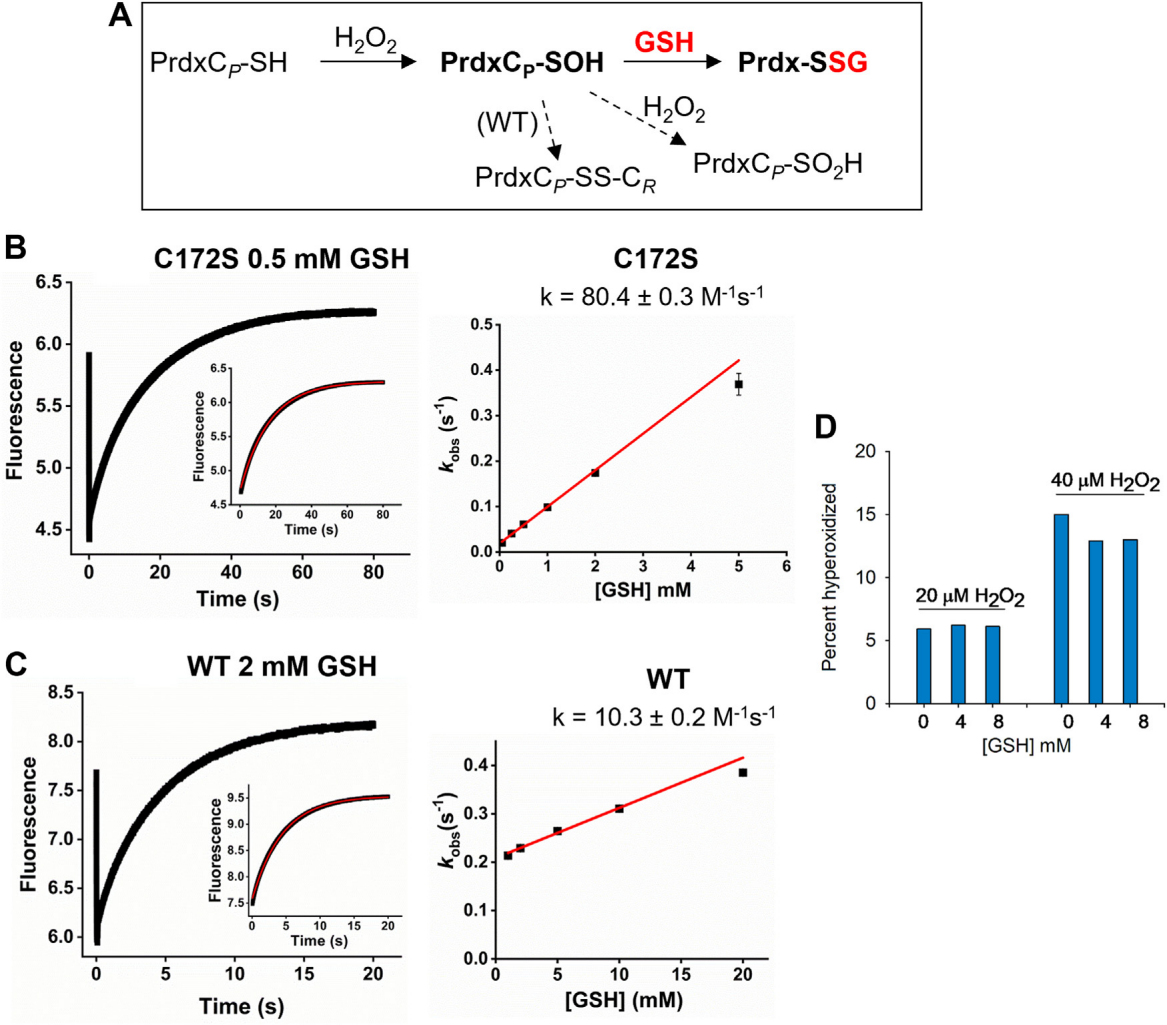
**Kinetics of condensation of GSH with the sulfenic acid of Prdx2.***A*, reactions under study (shown for one active site of Prdx2). *B* & *C*, stopped flow analysis for C172S and WT Prdx2 respectively measured with stated concentrations of GSH. Trp fluorescence (ʎ_ex_ = 280 nm, emission filter >320 nm) was measured on the rapid mixing of 0.5 μM reduced C172S Prdx2 (B) or WT Prdx2 (C) with H_2_O_2_ (1 μM) as described under Experimental procedures. This gives an initial decay phase due to C_P_–SOH formation, then a slower returning fluorescence due to disulfide formation (PrdxSSG for mutant; PrdxSSPrdx plus PrdxSSG for WT). Insets show single exponential fits of the returning fluorescence for *k*_obs_ calculation. *Right* panels show linear fits of the *k*_obs_ values obtained by single exponential fits *versus* GSH concentrations. Reactions were performed in 50 mM sodium phosphate buffer (pH 7.4; 25 °C). Data are representative of two independent experiments which gave second order rate constants of 80.4 ± 0.3 and 103 ± 37 M^−1^s^−1^ for the C172S mutant and 10.3 ± 0.2 and 4.0 ± 0.2 M^−1^s^−1^ for the WT protein. *D*, effect of GSH on hyperoxidation of WT Prdx2 as detected by quantitative whole protein MS analysis. Reduced Prdx2 was treated with stated concentrations of H_2_O_2_ in the presence and absence of GSH, then with DTT (10 mM) to convert oxidized and glutathionylated Prdx2 to the reduced form. LC/MS analysis was performed and calibrated with standard mixtures of reduced and hyperoxidized Prdx2 (as in Fig. S1). Each bar represents a single analysis.

Previously (5), we studied this reaction using the resolving Cys mutant of Prdx2, C172S, as this avoided the complication of competition with internal C_*P*_-C_*R*_ disulfide formation (see Fig. 2*A*). By measuring the ability of GSH to protect against hyperoxidation by excess H_2_O_2_ in the presence of various concentrations of GSH, we observed a rapid reaction between GSH and the sulfenic acid with a rate constant of approximately 250 M^−1^s^−1^. Here we have followed this reaction for the mutant and WT protein by monitoring changes in Trp fluorescence using stopped flow (7, 8). With a 2-fold excess of H_2_O_2_, C172S Prdx2 (Fig. 2*B*) showed the expected rapid loss in fluorescence due to oxidation of the C_*P*_ thiol to the sulfenic acid followed by a slower, GSH-dependent recovery reaction. The recovery phase we attribute to condensation of the sulfenic acid with GSH. Note that the recovery due to internal disulfide formation, as seen with the WT protein, cannot occur with the mutant (9). The recovery phase was fitted to a single exponential (inset) to give *k*_obs_ values. These were plotted against GSH concentration (right panel) to give a second order rate constant of 80 and 100 M^−1^s^−1^ for the two independent experiments. The reaction of the C172S mutant was also followed using MS to monitor the loss of the sulfenic acid in the presence of GSH, to give a rate constant of 550 M^−1^s^−1^ (Fig. S1*A*). Although MS gave higher values than the stopped flow, both show that the C172S mutant condenses rapidly with GSH.

Compared with the mutant, the sulfenic acid of WT Prdx2 was much less reactive with GSH (Fig. 2*C*). As reported previously (7, 8), stopped flow analysis of the reaction of reduced Prdx2 with H_2_O_2_ alone showed an initial decrease in fluorescence, then a second recovery phase due to condensation of C_*P*_-SOH with C_*R*_ to give the disulfide. Reaction with GSH was evident as a concentration-dependent increase in recovery rate. However, plotting exponential fits of this rise against GSH concentration (right panel) shows that this was modest and replicate experiments gave a second order rate constant (*k*_*1*_) of only 10 and 4 M^−1^s^−1^. The intercept of ∼0.24 s^−1^ corresponds to condensation with C_*R*_ and is in good agreement with literature values (7, 8). The difference between the WT and C172S proteins was unexpected considering their closely related crystal structures and similar rates of oxidation and hyperoxidation (9). However, the C172S mutant differs in other respects, including showing greater flexibility than the WT and being partially dissociated from the decameric state in its reduced form (9).

Further evidence for a slow reaction of WT Prdx2 was obtained using quantitative MS to test the ability of GSH to protect against Prdx2 hyperoxidation by excess H_2_O_2_. This was assessed by treating Prdx2 with H_2_O_2_ and GSH, reducing all the disulfide and glutathionylated species, then analyzing for reduced and hyperoxidized Prdx2. This method, which was calibrated using standards of each (Fig. S1*B*), avoided problems due to the various Prdx2 species differing in their MS response. As shown in Figure 2*D* for treatment with two concentrations of H_2_O_2_, no appreciable protection against hyperoxidation was apparent with up to 8 mM GSH.^1^ In contrast, GSH concentrations below 100 μM gave substantial inhibition with the C172S mutant (5). A rate constant for condensation of the sulfenic acid with C_*R*_ of 0.2 to 0.5 s^−1^ has been measured by stopped flow (7, 8). With a rate constant of 10 M^−1^s^−1^ for glutathionylation, this means that GSH concentrations well above 10 mM would be required to compete with disulfide formation. Hence our results show that glutathionylation by physiological GSH concentrations would be inefficient by this mechanism.

### Prdx2 glutathionylation by thiol disulfide exchange

**Figure 3:**
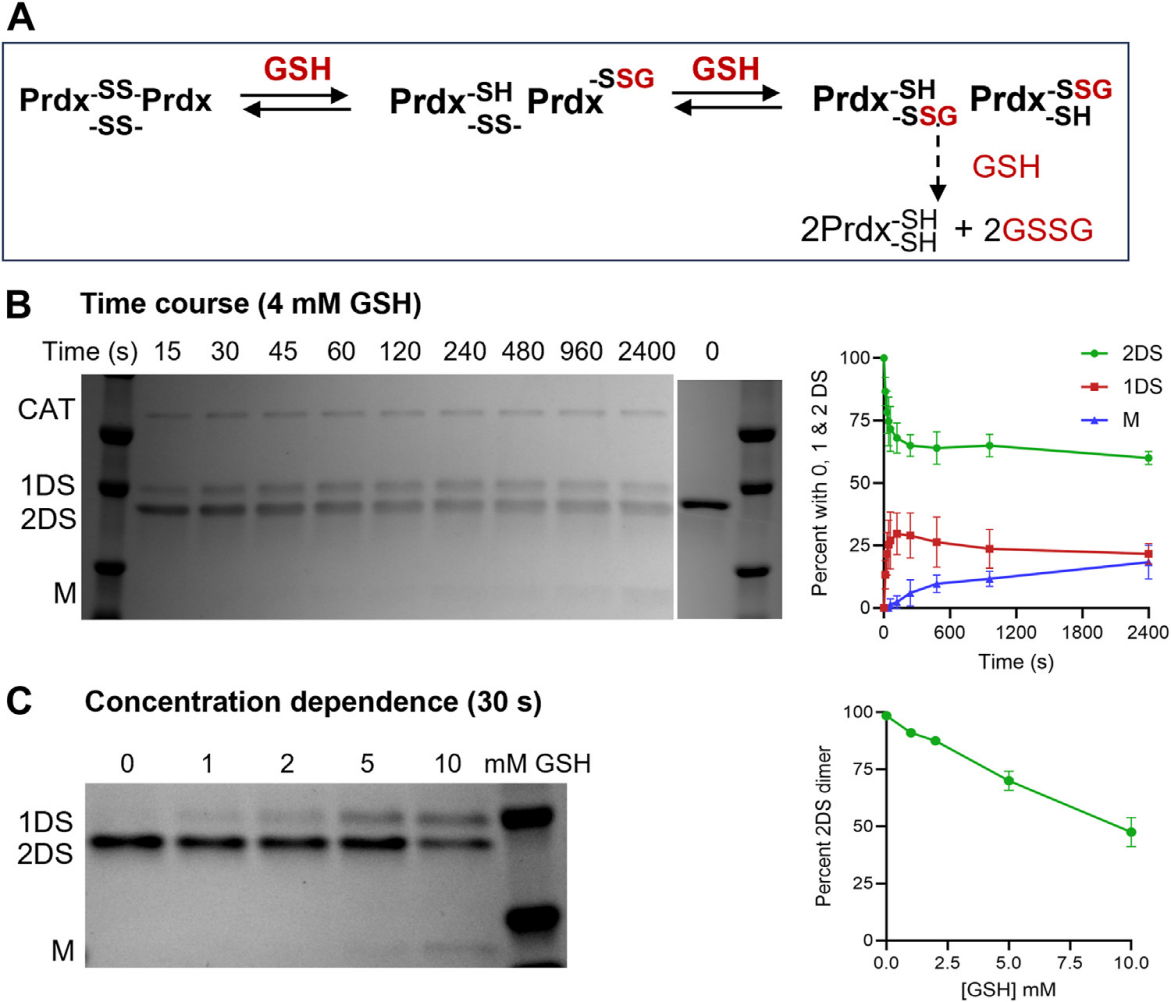
**Glutathionylation of oxidized Prdx2 by disulfide exchange.***A*, exchange reaction under study. *B*, time-dependent reaction of 5 μM Prdx2 disulfide with 4 mM GSH. Reaction was carried out in the presence of catalase (CAT, 20 μg/ml) and stopped at designated times with 20 mM NEM. Nonreducing SDS-PAGE shows progressive conversion of 2DS dimer to dimer with 1DS and then to monomer. This gel is from Figure S2*B* and has been used with gels from replicate experiments (Fig. S2, *A* and *C*) for kinetic modeling. The lane showing the zero time point is from the same experiment but run on a different gel. Densitometry measurements for the three replicates (means + SD) are shown in the *right* panel. Further analysis of the individual time courses is performed in the supplement with results described in Figure 7. *C*, GSH concentration dependence of exchange reaction with Prdx2 disulfide (5 μM) measured at 30 s in the absence of catalase. *Right* panel shows mean and range for the loss of 2DS dimer for two independent experiments. Molecular weight markers (*right* lane) are from the *bottom*, 25, 37, and 50 kDa (50 kD not shown for *C*). 1DS, covalent dimer with 1 disulfide; 2DS, dimer with 2 disulfides, M, reduced monomer.

As previously reported (5), Prdx2 glutathionylation can also occur by thiol-disulfide exchange between GSH and oxidized (disulfide-linked dimeric) Prdx2, as represented in Figure 3*A*. Here, we have analyzed this reaction in detail by nonreducing SDS-PAGE to gain a mechanistic understanding and kinetic data on the individual steps. As shown in Figure 3*B* (and replicates in Fig. S2), on mixing with GSH (plus catalase), the 2-disulfide dimer of Prdx2 (2DS) underwent time-dependent conversion to a slower-moving band corresponding to the 1-disulfide dimer (1DS), followed by gradual loss of both dimeric species and formation of monomer (M). Catalase was included to prevent the small amounts of adventitious H_2_O_2_ present in the solution accelerating the exchange process. This is evident from comparing Figs. 3 and S2 with a gel showing changes in the absence of catalase (Fig. S3) and is explained in the next section. Combined densitometry data from the three experiments (Fig. 3*B* right panel) shows that disulfide exchange was evident within seconds and stabilized to a pseudo-steady state situation within about a minute. Further kinetic analysis of the individual time courses to obtain rate and equilibrium constants is described in the kinetic modeling section below. The extent of exchange increased with increasing GSH concentration (Fig. 3*C*). Parameters obtained from modeling the data (see below) fit with increases in both the rate and the extent of glutathionylation at equilibrium.

The gel data were related to analyses of the glutathionylation state of the Prdx2 performed by MS. We previously showed using whole protein MS that oxidized Prdx2 treated with GSH produces monoglutathionylated products, that is, dimer with one —SSG linkage and one disulfide (1G-D), and subsequently monomer with one —SSG linkage and one reduced thiol (1G-M) (5). Here we determined the site of glutathionylation by performing chymotryptic digestion and peptide analysis. As shown for treatment with 4 mM GSH for 15 s, (Fig. S4), both C_*P*_ and C_*R*_ rapidly become partially glutathionylated during the initial stage of the reaction. These results are consistent with exchange reactions occurring at both Prdx2 active sites as depicted in Figure 3*A*.

### Prdx2 glutathionylation by GSH in the presence of H_2_O_2_

**Figure 4:**
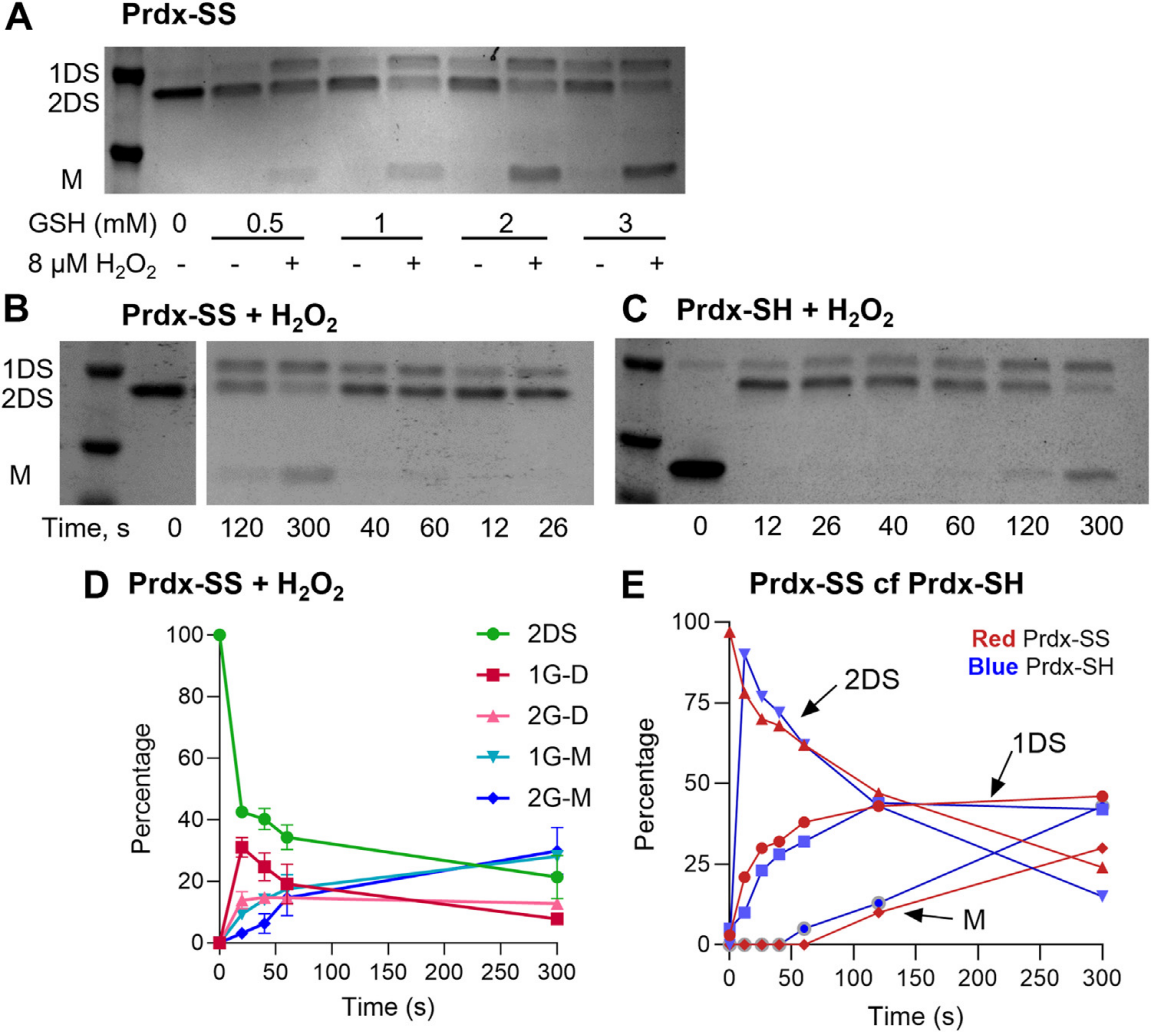
**Glutathionylation of Prdx2 in the presence of H_2_O_2_.***A*, nonreducing gel showing greater GSH concentration-dependent conversion of Prdx2 2DS dimers to 1DS dimers and monomers in the presence of 15 μM H_2_O_2_. Reaction was stopped at 10 min with 20 mM NEM. *B*, time course for the reaction of 5 μM Prdx2 disulfide with 8 mM GSH and 8 μM H_2_O_2_. *C*, time course for the reaction of 5 μM reduced Prdx2 with 8 mM GSH and 8 μM H_2_O_2_. Representative results are shown from an experiment in which reduced and oxidized Prdx2 were analyzed side by side. The lanes in (B) are from the same gel from which *middle* lanes have been excised. *D*, plots of densitometry (means, SD) from the four experiments shown in Figure S5*B*, with 8 mM GSH and 20, 50, 100, and 200 μM H_2_O_2_. *E*, densitometry traces from B (*red* symbols) and C (*blue* symbols) showing similar changes. Molecular weight markers in *left* lanes are from the *bottom*, 25 and 37 kDa; *B* & *C* also show a 20 kD marker. Gels from other experiments with Prdx disulfide carried out under comparable conditions and subjected to kinetic analysis are shown in Figure S5; a replicate time course with reduced Prdx2 is in FigureS6. Abbreviations as in Figure 3, plus 1-GD, covalent dimer with 1 GS adduct; 1-GM, monomer with 1 GS adduct; 2-GD, covalent dimer with 2 GS adducts; 2-GM, monomer with 2 GS adducts.

The mechanism of glutathionylation is more complex in the presence of H_2_O_2_, regardless of whether the starting material is the oxidized or reduced form of Prdx2. As shown by the SDS-PAGE profile in Figure 4*A*, when oxidized Prdx2 was incubated with a range of GSH concentrations, the GSH-dependent conversion to the 1DS and monomer bands was much greater in the presence of H_2_O_2_. Time course analyses of the reaction of oxidized Prdx2 with 8 mM GSH and H_2_O_2_ (Figs. 4*B* and S5) showed initial conversion from 2DS to 1DS and subsequent monomer formation that were both faster and more extensive than in the absence of H_2_O_2_. Experiments carried out with 10 to 200 μM H_2_O_2_ gave the same distribution of products in each case, as shown in Figure 4*D* for the plot of the mean densitometry data from Fig. S5*A*. Thus, H_2_O_2_ concentrations in this range were kinetically saturating, in the sense that concentrations in excess of ∼10 μM virtually do not influence the glutathionylation dynamics. More extensive kinetic modeling of these results is described below.

Treatment of reduced Prdx2 with a modest excess of H_2_O_2_ in the presence of GSH resulted in rapid, GSH concentration-dependent, conversion to 2DS dimers followed by sequential formation of 1DS dimers and monomers (Figs. 4*C* and S6). Higher H_2_O_2_ concentrations gave a similar profile but with progressively more hyperoxidation (as detected by MS, see below). The gels shown in Figure 4, *B* and *C* were obtained for oxidized and reduced Prdx2 treated identically in the same experiment. A comparison of the densitometry profiles from these gels (Fig. 4*E*) shows that after initial rapid conversion of the reduced protein to the 2DS dimer, they exhibit the same time course for conversion between the different bands. This is consistent with the evidence described above that condensation of the initially formed sulfenic acid has little impact on glutathionylation. It also means that provided hyperoxidation is taken into account, the kinetic and mechanistic data obtained for the oxidized protein can be applied to the reduced form.

**Figure 5:**
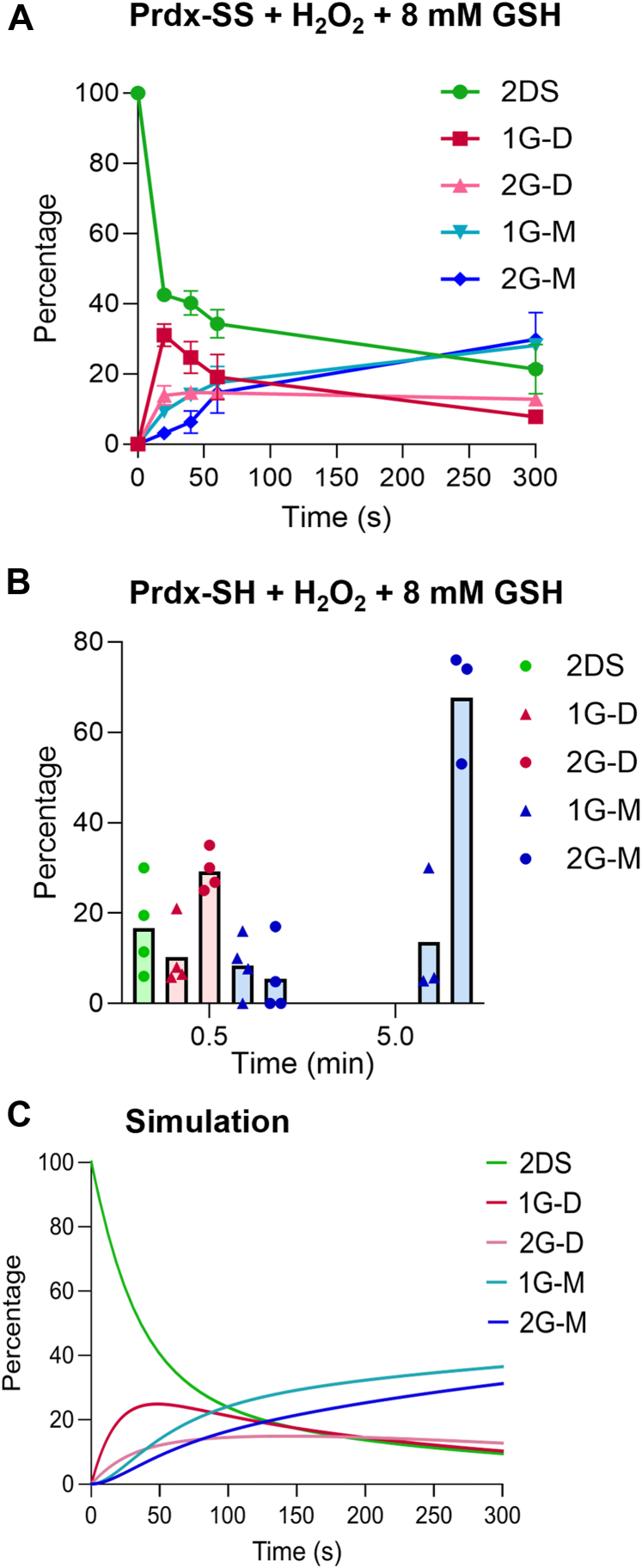
**Formation of glutathionylated products in the reaction of Prdx2 with GSH and H_2_O_2_.***A*, time course of the product formation from 5 μM Prdx2 disulfide reacted 8 mM GSH and H_2_O_2_. Reactions were stopped at stated times by adding catalase (20 μg/ml) and immediately diluting in acetonitrile/formic acid LC/MS running buffer. Results are means and averages of four analyses with H_2_O_2_ concentrations of 10, 20, 50, and 200 μM. Gels for the same samples are shown in Figure S5*B*. A contribution of <10% hyperoxidized protein, which was present in the controls and did not increase with treatment, has been subtracted before analysis. *B*, product distribution at 0.5 and 5 min after treatment of 5 μM reduced Prdx2 with 8 mM GSH and 20 μM H_2_O_2_. Reaction was stopped by adding catalase (20 μg/ml) followed immediately by 20 mM NEM. Treatment of reduced Prdx2 with 20 μM H_2_O_2_ alone caused hyperoxidation of the Prdx2 to give approximately 30% hyperoxidized dimer. As hyperoxidation was unaffected by GSH (Fig. 2*D*), this species has not been included in the distribution analysis. Results show data from three to four separate experiments. Products were analyzed by whole protein LC/MS and percent distributions are based on signal intensity. *C*, simulated time courses of product formation under the conditions of experiment in (*A*), based on the reaction network in Figure 7*F* and on the parameter estimates in Figure S15 obtained from the fit shown in Figure 7*G* to the SDS-PAGE densitometry data. Details of the modeling and parameter estimation are presented in SI3.2.2. 2DS, 2 disulfide dimer; 1G-D, dimer with 1 disulfide and 1 GSH attached; 2G-D, dimer with 1 disulfide and 2 GSH; 1G-M, monomer with 1 GSH; 2G-M, monomer with 2 GSH.

Whole protein MS was performed to detect glutathionylated products. Analyses were performed on oxidized Prdx2 treated as in Fig. S5*B* with H_2_O_2_ concentrations ranging from 10 to 200 μM in the presence of 8 mM GSH. As observed with the gel results, all H_2_O_2_ concentrations gave a similar product profile and the grouped data are shown in Figure 5*A*. In contrast to the monoglutathionylation seen with disulfide exchange in the absence of H_2_O_2_, in the presence of H_2_O_2_ both mono- and di-glutathionylated species were major products. The time course shows initial exchange at one disulfide to form mono- and di-glutathionylated dimers, then reaction at the other disulfide to form glutathionylated monomeric species as the major products. No hyperoxidized products were detected, even at high H_2_O_2_ concentrations. Because our analysis is based on signal intensity and different Prdx species give different MS responses, it does not allow absolute quantification. However, the temporal changes in profile of the glutathionylated products show a similar trend as for the conversion of gel bands.

MS analysis of reduced Prdx2 treated with a range of H_2_O_2_ and GSH concentrations was also performed. Figure 5*B* shows typical product distributions seen during the early stage of the reaction and after 5 min. The most notable difference between treating reduced and oxidized Prdx2 with H_2_O_2_/GSH was the progressive increase in hyperoxidation (sulfinic acid formation) with increasing concentration of H_2_O_2_ for the reduced protein. Consistent with previous observations (10), this was detected as hyperoxidized dimer. It accounted for ∼20% of the protein with 20 μM H_2_O_2_ (equivalent to the 10% value in Figure 2*D* as only half the sites in the dimer are hyperoxidized) and as described in Figure 2*D*, formed quickly, was not influenced by GSH and did not change over time. In contrast, no sulfinic acid was detected with oxidized Prdx2 and GSH, even at 200 μM H_2_O_2_. A comparison of the product distributions (excluding the hyperoxidized dimer) at 0.5 and 1 min for reduced Prdx2 (Fig. 5*B*) with the data in Figure 5*A* shows a similar trend in product profile to the oxidized protein, with initial conversion to mono- and di-glutathionylated dimers and subsequent conversion to glutathionylated monomers.

### Enhancement of C_*P*_ reactivity with GSH through modification of C_*R*_

**Figure 6:**
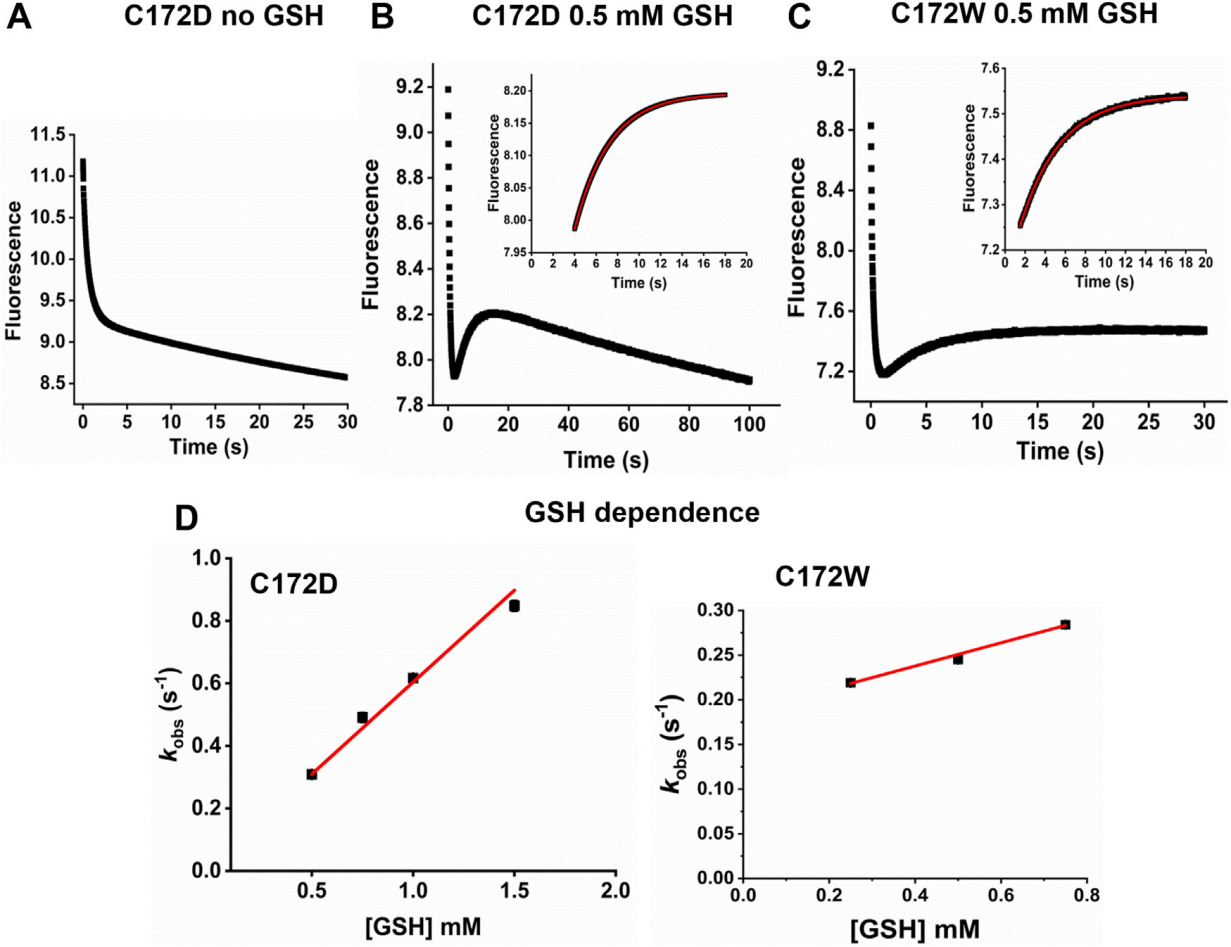
**Kinetics of glutathionylation of C172D and C172W mutants of Prdx2 in the presence of H_2_O_2_ measured by stopped flow.** Fluorescence changes of Prdx2 C172D + H_2_O_2_ (*A*) with no GSH or (*B*) with the addition of 500 μM GSH and (*C*) Prdx2 C172W + H_2_O_2_ with 500 μM GSH. Insets**:** Enlargement of time course of returning fluorescence with *red* lines showing single exponential fits. *D*, GSH dependence of *k*_obs_ calculated from returning fluorescence to give second order rate constants. Reactions were performed and intrinsic protein fluorescence was measured as in Figure 2 with prereduced Prdx2 mutants (1 μM), H_2_O_2_ (2 μM), and varying concentrations of GSH. Data are representative of two independent experiments which gave *k*_obs_ values for C172D of 590 ± 34 and 310 ± 8 M^−1^ s^−1^ and for C172W of 140 ± 14 and 130 ± 8.6 M^−1^s^−1^.

To explain the influence of H_2_O_2_ on Prdx2 glutathionylation, we are proposing that GSH reacts with C_*P*_-SOH when C_*R*_ is glutathionylated, even though this reaction is not favored for the native protein. In part, this can be explained by C_*R*_ being blocked and unable to form the disulfide, but modifying C_*R*_ could also result in enhanced reactivity of the sulfenic acid with GSH. Increased reactivity with GSH is suggested by the absence of detectable hyperoxidation when Prdx2 disulfide was treated with high H_2_O_2_ concentrations in the presence of GSH (see Fig. 5*A*). We were unable to generate pure Prdx2 with only C_*R*_ glutathionylated but based on the likely structural disruption caused by glutathionylation, we have mimicked the impact on C_*P*_ reactivity using two disruptive C_*R*_ mutants, namely C172D and C172W. These mutations were selected to introduce charge or bulk at the C_*R*_ site. They have been shown to cause partial dissociation of the decameric structure of the reduced protein and to decrease the rate of reaction of H_2_O_2_ with the reduced protein and the sulfenic acid each by more than 100-fold (9). This indicates that disruption in the C_*R*_ region caused by the mutations alters the active site structure that gives C_*P*_ its high reactivity with H_2_O_2_. To test whether the reactivity of the sulfenic acid with GSH is likewise affected, we followed the reaction using stopped flow. As shown in Figure 6*A*, treatment of 1 μM C172D, with a 2-fold excess of H_2_O_2_ alone caused relatively slow initial loss of fluorescence due to sulfenic acid formation, which is consistent with the low rate constant measured for this reaction (9), then a gradual drop due to photobleaching. With GSH, there was an additional second phase increase in fluorescence (Fig. 6*B*). The lower concentrations of GSH gave good concentration dependence with estimated rate constants for its reaction with C_*P*_-SOH from two independent experiments of 310 ± 8 and 590 ± 40 M^−1^s^−1^. The kinetics were more complex with C172W (Fig. 6*C*), perhaps because the fluorescence measurements were complicated by the additional Trp residue in the protein, and a linear dependence on GSH was found only up to 0.8 mM GSH. Data from duplicate experiments gave a condensation rate constant of 130 and 140 M^−1^s^−1^. The values for both mutants are much higher than measured for the WT protein (Fig. 2). Along with the increase seen with the C172S mutant, they support the concept that structural disruption at C_*R*_ causes a pronounced enhancement in the GSH reactivity of the sulfenic acid at C_*P*_.

### Kinetic modeling and parameter estimation

To gain further insight into the mechanisms of Prdx2 glutathionylation and estimate the attending rate and equilibrium constants, we resorted to kinetic modeling and fitting to time courses from densitometry of SDS-PAGE experiments described above. We first analyzed the early time courses of 2DS dimer fractions in experiments where oxidized Prdx2 was incubated with GSH in the presence of either catalase or kinetically saturating H_2_O_2_. These analyses drew on kinetic models that yield tractable analytical solutions, which facilitated mechanistic interpretation. We then leveraged on the insights thus gained to analyze the time courses of the other dimer and monomer fractions, seeking to evaluate the extent to which the state of one active site influences the reactivity of the other one in the same dimer. Modeling assumptions, descriptions of the models, results, and discussions are presented in detail in the Supporting Information Section 3 (SI3) and more summarily below. A list of symbols used is given at the end of the article.

**Figure 7:**
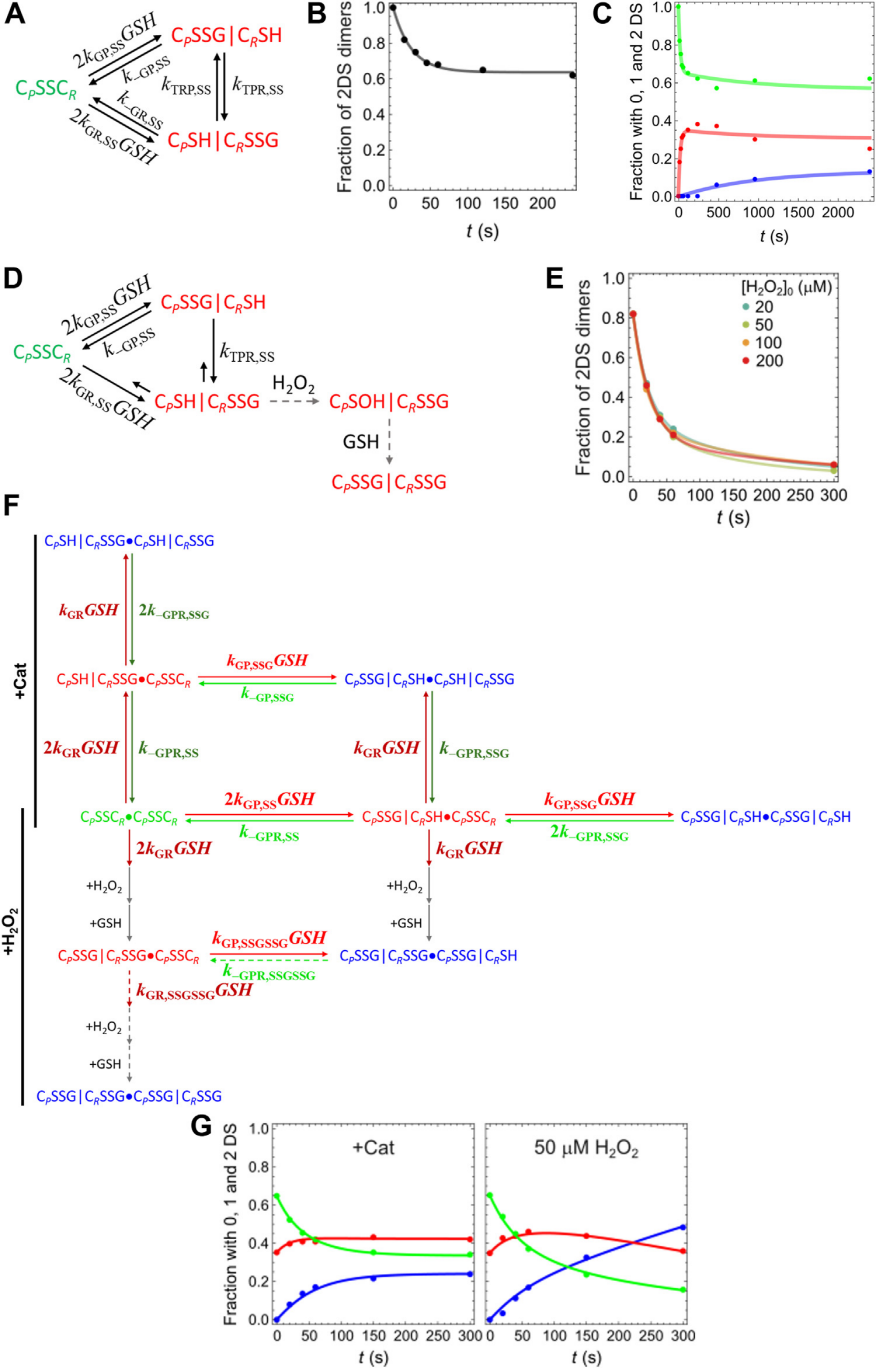
**Modeling and quantitative analysis of the formation of glutathionylated products in the reaction of Prdx2 with GSH and H_2_O_2_.***A*, reaction scheme underlying the model to fit the decay of the fraction of 2DS Prdx2 dimers in the presence of GSH and catalase. The (disulfide) state of the second active site in the dimer is omitted for simplicity. The species represented by *green* and *red* symbols run as 2DS and 1DS dimers in nonreducing SDS-PAGE gels, respectively. *B*, curve fit to the time course of the fraction of 2DS dimers in Figure 3*B*, replicated from Figure S8*B*. *C*, curve fits of the fraction of dimers and monomers in Figure 3*B*, replicated from Figure S13*B*. *D*, reaction scheme underlying the model to fit the early decay of the fraction of 2DS Prdx2 dimers in the presence of GSH and kinetically saturating H_2_O_2_ concentrations. The model does not explicitly consider the reactions in *dashed gray*, which are shown just to indicate that glutathionylation at C_*P*_ and C_*P*_ → C_*R*_ GS transfer are considered irreversible due to the strong competition of sulfenylation with the respective reverse reactions. The (disulfide) state of the second active site in the dimer is omitted for simplicity. *E*, curve fits to the time courses of the fraction of 2DS dimers in the gel analyses of Prdx2 disulfide treated with 5 mM GSH and various concentrations of H_2_O_2_ as depicted in Figure S5*A*, replicated from Figure S9*A*. *F*, reaction scheme underlying the model to fit the time courses of the fractions of Prdx2 2DS dimers, 1DS dimers, and monomers in the presence of GSH and zero or kinetically saturating H_2_O_2_ concentrations. The “+Cat” and “+H_2_O_2_” parts of the reaction network are considered operative only in the presence of catalase or in the presence of saturating H_2_O_2_, respectively. The reactions indicated by *dashed* arrows were neglected in the final model, and those indicated by *gray arrows* were not explicitly considered because the overall processes in the sequences are rate limited by the first steps. Glutathionylation (*red arrows*) and deglutathionylation (*green arrows*) reactions involving C_*R*_ are indicated in darker shades than the corresponding reactions involving C_*P*_. *G*, curve fits to the time courses of the fraction of dimers and monomers in the gels in Figure S5*B* (+Cat and 50 μM H_2_O_2_), replicated from Figure S15*A*. In (*A*) and (*D*), *green*, *red*, and *blue* symbols indicate species running as 2DS dimers, 1DS dimers, and monomers (respectively) in nonreducing SDS-PAGE gels.

To estimate equilibrium and rate constants for Prdx2 glutathionylation and deglutathionylation through thiol-disulfide exchange, we fitted a simple kinetic model to the first 4 min of the decay of the fraction of dimeric units with two disulfides (*f*_2_) obtained from the densitometry of the gels in Figs. 3*B* and S2. No monomers were detected during this time period, which allowed to focus on the events at a single active site without concern for the effects of changes in the other site in the same dimeric unit. Thus, the model was based on the reaction scheme in Figure 7*A*. As explained in SI3.1, this model predicts a bi-exponential decay, whereas a strictly mono-exponential decay (as in Fig. 7*B*) was observed. The latter might be due to either the rate constants for self-deglutathionylation from C_*P*_ and from C_*R*_ having nearly identical values or to very fast equilibration of one of the three thiol-disulfide exchange reactions. Subsequent analysis of the dynamics of glutathionylation in the presence of H_2_O_2_ (below and SI3.1) supported the former explanation and not the latter. Consequently, the dynamics of the decay simplifies to

(3) $$f_{2}\left(t\right)=f_{2}\left(0\right)\frac{k_{-GPR,SS}+2k_{G,SS}GSH\;e^{-\left(k_{-GPR,SS}+2k_{G,SS}GSH\right)t}}{k_{-GPR,SS}+2k_{G,SS}GSH}$$

where *k*_*G*,*SS*_ = *k*_*GP*,*SS*_ + *k*_*GR*,*SS*_ and *k*_−*GPR*,*SS*_ = *k*_−*GR*,*SS*_ = *k*_−*GP*,*SS*_ are the glutathionylation and deglutathionylation rate constants, respectively. The excellent fits of this expression to the data from three independent experiments yielded the best-fit estimates shown in the two leftmost columns of Table 1 (Fig. 7*B*, more details in Fig. S8).

**Table 1:** Estimates from fits to the densitometry of SDS-PAGE gels following the time courses of glutathionylation after incubation of oxidized Prdx2 with 8 mM GSH in the presence of catalase

Fit to *f*_2_ (*n* = 3)		Fit to *f*_0_, *f*_1_, *f*_2_ (*n* = 3)	
Parameter	Mean ± SEM	Parameter	Mean ± SEM
***k*_*G,SS*_ (M^−1^s^−1^)**	**1.45 ± 0.06**	*k*_*G,SS*_ (M^−1^s^−1^)	*1.7 ± 0.3*
***k***_***−GPR,SS***_**(s^−1^)**	**0.0208 ± 0.0012**	***k***_***−G,SS***_**(s^−1^)**	**0.030 ± 0.003**
*K*_*−G,SS*_ (mM)	*14.3 ± 1.0*	***K***_***−G,SS***_ (mM)	**21. ± 3.**
		*k*_*G,SSG*_ (M^−1^s^−1^)	*0.6 ± 0.3*
		*k*_*−G,SSG*_ (s^−1^)	*(2. ± 1.) × 10*^*−3*^
		*K*_*−G,SSG*_ (mM)	*4.1 ± 0.1*
		***R***_***−G,SSG***_	**0.22 ± 0.03**
		***r***_***−G,SSG***_	**0.11 ± 0.05**

Adjustable parameters in each set of fits are shown in bold; values of derived quantities are shown in italics. Results in the two left hand and in the two right hand columns are from fits to the first 4 min and 40 min of incubation, respectively. The number of independent experiments is shown in parentheses in the header row. Estimates from individual runs and experiments are shown in Figs. S8 and S13. Symbols are defined at the end of the article.

Modeling of the reaction between oxidized Prdx2, GSH, and H_2_O_2_ yields further insights. A model based on the scheme in Figure 7*D* (Model 1 in SI3.1) predicts that under these conditions, *f*_2_(*t*) shows a bi-exponential decay defined by the aggregated parameters *γ*=*k*_*G*,*SS*_, *δ*=*k*_*−GP,SS*_+*k*_*TPR,SS*_ and *θ*=*k*_*GP,SS*_*k*_*−GP,SS*_. This model does indeed excellently fit the densitometric readings from the gels in Figs. 4*B* and S5, *A*–*C*, as shown in Figs. 7*E* and S9, and provides tight estimates for these three parameters (Table 2). Although these estimates do not uniquely determine the values of the four mechanistic parameters (*k*_*GP*,*SS*_,*k*_−*GP*,*SS*_,*k*_*GR*,*SS*_,*k*_*TPR*,*SS*_), they provide useful constraints for them, as derived in SI3.1. These are shown in the two left hand columns of Table 2, with further detail in Table S1.

**Table 2:** Estimates and bounds from fits to the densitometry of SDS-PAGE gels following the time courses of glutathionylation after incubation of oxidized Prdx2 with 8 mM GSH in the presence of H_2_O_2_

Fit to *f*_2_ (*n* = 2)		Fit to *f*_0_, *f*_1_, *f*_2_ (*n* = 2)	
Parameter	Mean ± SEM	Parameter	Mean ± SEM
***γ* = *k*_*GR,SS*_ + *k*_*GP,SS*_ (M^−1^s^−1^)**	**1.770 ± 0.008**		
***δ*** = ***k***_***−GPR,SS***_ + ***k*_*TPR,SS*_ (s^−1^)**	**0.01475 ± 0.000027**		
***θ* = *k*_*GP,SS*_*k***_***−GP,SS***_**(M^−1^s^−2^)**	**0.01306 ± 0.00035**		
*k*_*GP,SS,min*_ (M^−1^s^−1^)	*1.170 ± 0.035*	***k***_***GP,SS***_**(M**^**−1**^**s**^**−1**^**)**	**1.05 ± 0.05**
*k*_*GP,SS,max*_ (M^−1^s^−1^)	*1.770 ± 0.008*		
*k*_*−GP,SS,min*_ (s^−1^)	*0.00865 ± 0.00021*		
*k*_*−GP,SS,max*_ (s^−1^)	*0.01475 ± 0.00027*	***k***_***−GPR,SS***_**(s**^**−1**^**)**	**0.0172 ± 0.0009**
*K*_*−GP,SS,min*_ (mM)	*4.36 ± 0.11*		
*K*_*−GP,SS,max*_ (mM)	*11.8 ± 0.5*	*K*_*−GP,SS*_ (mM)	16.54 ± 0.33
*k*_*GR,SS,max*_ (M^−1^s^−1^)	*0.72 ± 0.04*	***k*_*GR*_ (M^−1^s^−1^)**	**0.286 ± 0.011**
*K*_*−GR,SS,min*_ (mM)^a^	*20.0 ± 1.2*	*K*_*−GR,SS*_ (mM)	*60. ± 4.*
(*k*_*GP,SS*_/*k*_*GR,SS*_)_min_	*1.58 ± 0.10*	*k*_*GP,SS*_/*k*_*GR,SS*_	*3.21 ± 0.23*
*k*_*TPR,SS,max*_ (s^−1^)	*0.00556 ± 0.00034*		
(*k*_*TPR,SS,max*_/*k*_*−GP,SS*_)_max_	*0.61 ± 0.05*		
*K*_*TRP,SS,min*_^a^	*1.58 ± 0.10*		
(*k*_*TRP,SS*_ + *k*_*−GR,SS*_)_min_ (s^−1^)^a^	*0.01475 ± 0.00027*		
		***k*_*GP,SSG*_ (M^−1^s^−1^)**	**1.03 ± 0.06**
		***k***_***−GPR,SSG***_**(s^−1^)**	**0.0093 ± 0.0006**
		*K*_*−GP,SSG*_ (mM)	*8.61 ± 0.23*
		*R*_*−GP,SSG*_	*0.55 ± 0.05*
		*r*_*GP,SSG*_	*0.99 ± 0.08*
		*r*_*−GPR,SSG*_	*0.55 ± 0.05*
		***k*_*GP,SSGSSG*_ (M^−1^s^−1^)**	**0.554 ± 0.025**
		*r*_*GP,SSGSSG*_	*0.481 ± 0.032*

Adjustable parameters in each set of fits are shown in bold; values of derived quantities are shown in italics. The number of independent experiments is shown in parentheses in the header row. Estimates from individual runs and experiments are shown in Tables S1, S2 and Figure S15.

a Assuming *k*_*−GR,SS*_ = *k*_*−GP,SS*_.

The estimates above show the following notable features, discussed in detail in SI3.1. (i) Glutathionylation and deglutathionylation of Prdx2 disulfides by thiol-disulfide exchange occur with rate constants ≈1.5 M^−1^s^−1^, and 0.02 s^−1^, respectively. Thus, the first equilibrium with 8 mM GSH establishes within about 0.5 min. (ii) C_*P*_ is preferred over C_*R*_ for glutathionylation, both kinetically (*k*_*GP*,*SS*_>1.6×*k*_*GR*,*SS*_) and thermodynamically (*K*_*TRP*,*SS*_>1.6) (Table 2). As explained in SI3.1, a moderate preference for C_*P*_ is also suggested by the peptide analysis in Fig. S4*B*. (iii) Based on the analysis of model 2 in SI3.1, the direct transfer of the glutathionyl moiety between C_*P*_ and C_*R*_ is slow, with an estimated half-life of >125 s. (iv) Due to the differences in the glutathionylation rate constants highlighted in point (i), the equilibrium constant for deglutathionylation from C_*P*_ (4.4–12 mM) is in the physiological GSH concentration range, whereas that for deglutathionylation from C_*R*_ exceeds 18 mM (Table 2). (v) The rate constant for sulfenylation of C_*P*_-SH by H_2_O_2_ at the C_*R*_-glutathionylated active site exceeds 750 M^−1^s^−1^. Though a conservative underestimate, this value highlights that the C_*P*_-SH retains a substantial reactivity despite the strong distortion of the active site expected from glutathionylation at C_*R*_. For comparison, values of ∼10^6^ M^−1^s^−1^ (100-fold less than for WT) have been measured for the disruptive C172D and C172W mutants (9).

To determine whether Prdx2 glutathionylation shows the same kinetic characteristics at each active site or whether glutathionylation at one site influences glutathionylation at the other, we next modeled the dynamics of the fractions of 1DS and 2DS dimers and of monomers. We first analyzed data from incubations of oxidized Prdx2 with GSH in the presence of catalase (Figs. 3*B* and S2), for which we considered the scheme in Figure 3*A*. To avoid a proliferation of nonidentifiable parameters, this model did not discriminate between C_*P*_ and C_*R*_ glutathionylation and considered a single GSH-mediated deglutathionylation step. The resulting reaction scheme leads to a kinetic model (model 3 in SI3.2.1) for the evolution of the fractions of dimeric units with two, one, or zero disulfides (*f*_2_,*f*_1_,*f*_0_, respectively, the latter running as monomers in SDS-PAGE gels). As detailed in SI3.2.1, the time courses of these fractions were computed by numerical integration and fitted to the first 40 min of the experimental time courses from the three independent experiments (Figs. 3*B* and S2) as in Figures 7*C* and S13. The obtained best-fit estimates are shown in the two righthand columns of Table 1, with further details in Fig. S13. (These results will be discussed below and more extensively in SI3.2.1.)

To gain further insight, we modeled the reaction of oxidized Prdx2 based on the complex scheme in Figure 7*F*. We made the following four simplifying assumptions, which are justified in SI3.2.2. (i) Glutathionyl transfer between C_*P*_ and C_*R*_ is negligible. (ii) Diglutathionylation of an active site in the presence of saturating H_2_O_2_ is rate limited by the release of C_*P*_-SH upon glutathionylation at C_*R*_. (iii) The rate constants for self-deglutathionylation of C_*P*_ and C_*R*_ are identical. (iv) Monoglutathionylation at C_*P*_ or at C_*R*_ has the same effect on the properties of the other site in the same dimeric unit. The numerical *f*_0_(*t*),*f*_1_(*t*),*f*_2_(*t*) solutions of the resulting model were globally fitted to pairs of time courses (one for the system with catalase addition, the other with a saturating H_2_O_2_ concentration) of the densitometric readings from the gels shown in Figs. S2*C* and S5, *B* and *C*. Preliminary fits consistently suggested that (v) diglutathionylation at one active site prevents glutathionylation at C_*R*_ and deglutathionylation from C_*P*_ at the other site and that (vi) whether one active site is in disulfide form or monoglutathionylated does not affect the rate constant for glutathionylation at C_*R*_ at the other site. A reduced model (Model 4 in SI3.2.2) embodying these two additional assumptions yielded good fits (Figs. 7*G* and S15) and tight estimates for the six adjustable parameters (Table 2, right hand columns, details in Fig. S15). Despite the model being derived from the gel data and the temporal changes in the different glutathionylated species measured by MS, the close agreement between the measured (Fig. 5*A*) and simulated changes in glutathionylation (Fig. 5*C*) is strong validation of the model and the kinetic estimates derived from it.

Overall, the modeling results for both active sites (two right hand columns of Tables 1 and 2) support the following conclusions. (i) Glutathionylation at one active site stabilizes the glutathionylated product at the other, as follows from the low *R*_-*G*,*SSG*_ and *R*_-*GP*,*SSG*_ values in Tables 1 and 2. (ii) This thermodynamic stabilization mainly reflects a slower self-deglutathionylation when both active sites are glutathionylated than when the other site is a disulfide. This conclusion follows from the low *r*_-*G*,*SSG*_ and *r*_-*GP*,*SSG*_ values in Tables 1 and 2, the glutathionylation rate constant being virtually insensitive to glutathionylation of the other site. (iii) Diglutathionylation at one site slows down glutathionylation of C_*P*_ of the other one, as *r*_*GP*,*SSGSSG*_<1 in Table 2. (iv) GSH-mediated deglutathionylation is very slow, as only one of the three fits in Fig. S13 yielded a significant but small rate constant for this reaction. This is consistent with previously reported MS analysis (5) showing most of the monomeric species glutathionylated and no observable 1DS dimer with no GSH in this time range.

The results also explain why the presence of H_2_O_2_ accelerates glutathionylation and monomer formation. The observed time course of the relative amounts of 2DS dimers, 1DS dimers, and monomers reflects a balance between opposing processes: the opening of the disulfide bonds by glutathionylation and their restoration by self-deglutathionylation. In the absence of H_2_O_2_, these processes equilibrate within a minute. In the presence of saturating H_2_O_2_, this equilibration dominates the early time course (Fig. S16). However, according to the estimates in Table 2, 21% (*k*_*GR*_/(*k*_*GR*_+*k*_*GP*,*SS*_)) of the 2DS dimers and 8.3% (*k*_*GR*_/(*k*_*GR*_+*k*_−*GP*,*SS*_+*k*_*GP*,*SSG*_)) of the C_*P*_-glutathionylated dimers proceed to C_*R*_-glutathionylation, and are then readily sulfenylated and diglutathionylated. These processes irreversibly block the restoration of the disulfides by self-deglutathionylation and thereby accelerate the *net* decrease of 2DS dimers and accumulation of monomers.

## Discussion

We showed previously that the active site cysteines of Prdx2 readily undergo glutathionylation and that GSH in combination with Grx is able to recycle oxidized Prdx2 and provide an alternative to the Trx/Trx reductase system (5). The glutathionylation reaction is complex, however, and depending on conditions gives rise to a range of mono- and di-glutathionylated species. Here we have investigated the kinetics and mechanism of glutathionylation and assessed the contributions of thiol/disulfide exchange with Prdx disulfide and condensation with Prdx-SOH to the process. This has produced some unexpected findings.

First, as measured by stopped flow, the condensation reaction had a surprisingly low rate constant of ∼10 M^−1^s^−1^. Consistent with this, millimolar GSH concentrations competed poorly with Prdx2 disulfide formation and were not effective at protecting against hyperoxidation. As these concentrations lie in the physiological range, glutathionylation *via* this mechanism should not be a favorable intracellular reaction. Prdx2 undergoes a typical 2Cys redox cycle involving an oxidation, condensation, and resolution step. Compared with the other steps, the rate of condensation of C_*p*_-SOH with C_*R*_-SH for Prdx2 is slow. Therefore, it might have been expected that this would provide a favorable situation for the sulfenic acid to react with GSH. However this is not the case.

For GSH and other low MW thiols, condensation between the sulfenic acid and thiol is very fast, with rate constants >10^5^ M^−1^ s^−1^ at neutral pH (11). Data on proteins is limited, with rate constants ranging from 2.9 M^−1^ s^−1^ for the buried, stable sulfenic acid of human albumin (12) to >10^5^ M^−1^ s^−1^ for plasmodium antioxidant protein, a 1Cys Prdx that uses GSH as its reducing substrate (13). Prdx2 appears to be at the low end of the scale. Thus, slow condensation with C_*R*_ is reflected in slow condensation with GSH, suggesting that common structural features limit the reactivity of C_*p*_-SOH with both substrates. The restriction for internal disulfide formation is attributed to transformation from the fully folded to locally unfolded Prdx2 structure and physical release of C_*p*_-SOH from the active site environment (14). This may also restrict reactivity with other thiols.

We found that condensation with GSH is substantially slower for WT Prdx2 than for the C172S variant. A higher rate constant for the mutant measured by stopped flow, direct monitoring of sulfenate loss in the presence of GSH, and the ability of GSH to protect only the mutant against hyperoxidation all support this conclusion. This is despite the close alignment between the crystal structures of WT and mutant protein (9). However, there are subtle increases in flexibility that presumably facilitate the release of C_*P*_-SOH from the active site and account for the difference. Consistent with this interpretation, the more disruptive C172D and C172W mutations, which decrease reactivity of C_P_-SH and C_P_-SOH with H_2_O_2_ by ∼100 -fold (9), were both even more reactive than C172S Prdx2 with GSH. Both mutations also caused substantial dissociation of the reduced protein from a decameric to dimeric quaternary structure (9) and these results imply that disruption of the structure by glutathionylation of C_*R*_ would also decrease reactivity of C_P_-SOH with H_2_O_2_ and increase reactivity with GSH. The differences in structure and reactivity raise a note of caution against using resolving Cys mutants such as C172S as surrogates for WT Prdxs. They may aid detection of binding partners by preventing the resolution step but will also produce a sulfenic acid that could undergo reactions that would not be favored with WT.

**Figure 8:**
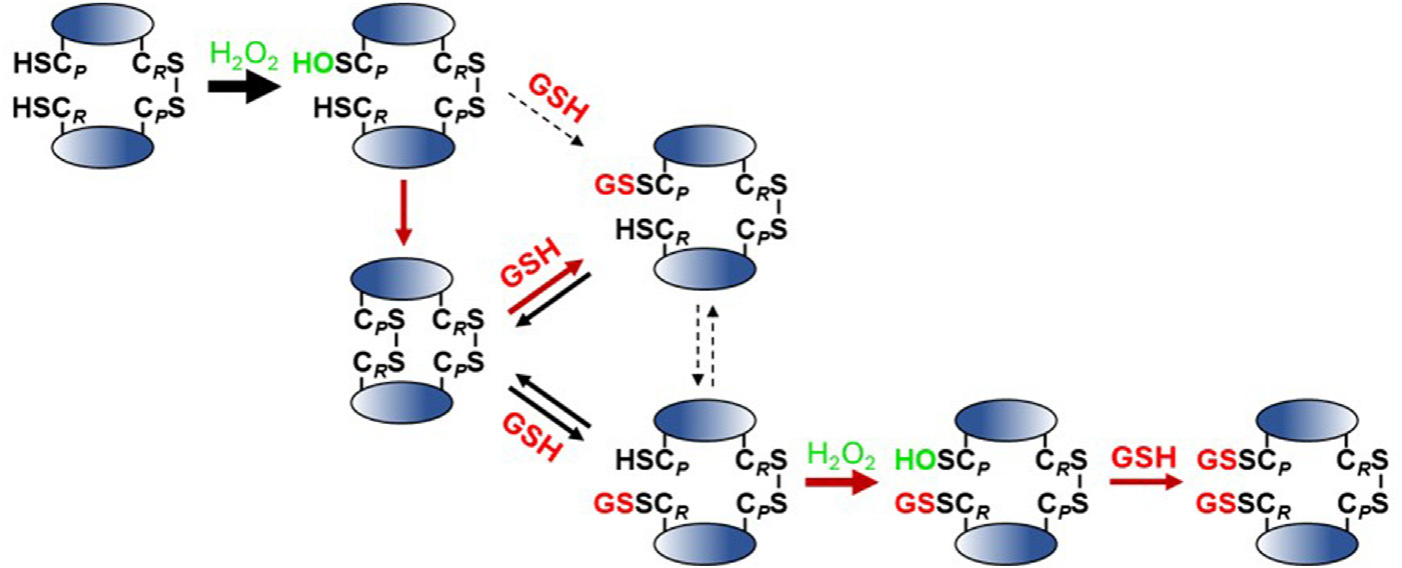
**Schematic of the mechanism of glutathionylation of reduced or oxidized Prdx2 by GSH in the presence of H_2_O_2_. Reactions at one active site are shown for simplicity but equivalent reactions also occur at the second active site.** Major reactions are shown in bold *red* arrows and those with dashed arrow are of only minor significance.

Taken together, our results fit with the mechanism depicted in Figure 8 in which thiol disulfide exchange, rather than condensation with C_P_-SOH, is the favored route to Prdx2 glutathionylation, regardless of the presence of H_2_O_2_. In the absence of H_2_O_2_, this proceeds by as a series of equilibria leading initially to monoglutathionylation at one site of the dimer and progressing to monoglutathionylation at the other. However, once C_*R*_ is glutathionylated, reaction of C_P_-SH with H_2_O_2_ and condensation of GSH with the C_P_-SOH so formed becomes important. This outcompetes further hyperoxidation by H_2_O_2_, displaces the equilibria, and promotes the formation of diglutathionylated dimeric and monomeric species.

Even though the rate constants for the exchange reactions are not particularly high, they are sufficient for equilibration with physiological GSH concentrations to occur within a minute.

This equilibration gives only partial conversion to glutathionylated dimer. Reduction of this dimer *via* thiol-disulfide exchange with GSH is very slow. Grx1 catalyzes this reaction (5), which should happen *in vivo*. The results presented in Figure 5*B* (5) suggest less effective catalysis than the very rapid reduction of Prdx2 disulfides observed with Trx1 (15). Moreover, the cytosolic Grx1 concentration in human cells is on average one order of magnitude lower than that of Trx1 (15). This would explain why oxidized Prdx2 can accumulate in cells with plenty of GSH (16, 17). Assuming that the rate-limiting step in Prdx2 disulfide reduction by GSH *in vivo* is the initial thiol-disulfide exchange reaction (k ∼ 1.5 M^−1^s^−1^, Table 1) allows the following quantitative comparisons. In hepatocytes carrying ∼8 mM GSH (18) and ∼63 μM Trx1 (15), reduction by fully reduced Trx1 is 3200-fold faster than by GSH, considering a 6.1 × 10^5^ M^−1^s^−1^ rate constant (15) for the former reaction. Moreover, their estimated 50 μM s^−1^ (16) Trx reductase activity makes it likely that turnover by even low concentrations of reduced Trx will predominate. However, the increased demands of oxidative stress can result in Trx becoming oxidized (19) and in diversion of Trx reductase to other targets. Oxidized Prdx accumulates in these situations and reduction *via* GSH should occur. This is likely to be particularly significant in erythrocytes. These cells carry ∼3 mM GSH (20) and 0.56 μM Trx1 (21) and although this means that Prdx2-SS reduction by fully reduced Trx1 is ∼76-fold faster than by GSH, their modest Trx reductase activity (estimated at ∼1.5 uM s^−1^ (22)) implies that Trx1 may be oxidized to the point where GSH significantly contributes to Prdx2 reduction. But irrespective of GSH's relative contribution for the reduction of the cytosolic 2-Cys Prdx, this process places a considerable oxidative load (∼1 μM s^−1^) on the GSH pool whenever the Prdx become substantially oxidized. The ability of GSH/Grx1 to provide an important backup for Prdx2 reduction by Trx is in agreement with the recent report that the yeast Prdx, TSA1, can be recycled by GSH/Grx and is a major contributor to GSSG formation, but this occurs subsequent to oxidation of Trx (23).

The exchange reaction is more complex when H_2_O_2_ is present, for both reduced and oxidized Prdx2. For the reduced protein, the initial step is oxidation by H_2_O_2_ to the disulfide, then disulfide exchange proceeding in the same way regardless of the starting Prdx2 oxidation state. The increased rate of conversion to glutathionylated products plus the formation of di- as well as mono-glutathionylated dimeric and monomeric species caused by even low concentrations of H_2_O_2_ can be explained by glutathionylation of C_*R*_ producing free C_*P*_-SH. This then reacts with H_2_O_2_ to give the sulfenic acid and subsequent condensation with GSH. The latter reaction will be enabled because condensation with derivatized C_*R*_ is not possible. Also, our results imply that this reaction would also be enhanced by C_*R*_ glutathionylation. At the typically lower H_2_O_2_ concentrations experienced *in vivo*, release of C_*R*_-SSG adduct by the action of Grx or by spontaneous attack by the C_*P*_-SH (*t*_1/2_≈40 s) before sulfenylation of the latter thiol should limit the formation of diglutathionylated species.

Our findings are relevant to Prdxs acting as transmitters of redox relays. This process, in which a Prdx acts as a sensor for an oxidant such as H_2_O_2_ and passes its oxidizing equivalents to another thiol protein, has gained favor as a cell regulatory mechanism in signal transduction (2, 3, 4, 24). A number of relays have been characterized (25, 26, 27, 28) and they provide an attractive solution for overcoming the low oxidant sensitivity of many regulatory proteins (28) and a way of achieving selectivity. Nevertheless, there are only a few well-defined examples. Oxidation of the target protein by the Prdx is proposed to occur either by condensation with the sulfenic acid or disulfide exchange to form an initial mixed disulfide, a reaction analogous to glutathionylation. Our findings on Prdx2 glutathionylation raise issues for both mechanisms. First, the limited ability of GSH to condense with the sulfenic acid in competition with internal disulfide formation implies that condensation with protein thiols would be ineffective unless there was some facilitating factor. Furthermore, disulfide exchange with GSH is driven kinetically and thermodynamically by high millimolar GSH concentrations. It should be much less efficient for protein thiols present at orders of magnitude of lower concentrations, unless promoted by some favorable driving force. Another potential constraint relates to the second step in a relay that releases the oxidized target. This requires attack by a second protein thiol (or GSH) to release the oxidized target and reduced Prdx. Yet GSH releases GSSG from the mixed disulfide to only a limited extent, favoring the stability of Prdx2-target mixed disulfide. This suggests that attack of a second target thiol to release a target homodisulfide could be unfavorable as well. In turn, the finding that derivatization of Prdx2 C_*R*_ allows C_*P*_-SH to retain a substantial reactivity with H_2_O_2_ and makes the ensuing C_*P*_-SOH more available suggests the possibility that this could facilitate condensation with targets. However, such targets would still have to compete with much more abundant GSH.

So how might a Prdx2-mediated relay be transmitted? A clue to one plausible mechanism comes from the finding (29) that an additional scaffold protein is required to facilitate the Prdx-mediated activation of the yeast transcription factor YAP1 (25) and the transcription factor STAT3 (30). For YAP1, the scaffold not only brings the reacting partners together but also influences the reactivity of the sulfenic acid to favor mixed disulfide as against internal disulfide formation. This may have more widespread applicability. Another possibility is that the Prdx does not react directly with the target but *via* Trx or a Trx-like intermediary. Trx interacts with a wide range of thiol proteins and although this is normally considered as a reductive process, its reactions are reversible and thus capable of promoting oxidation of regulatory proteins (31). While our study has focussed on Prdx2, it has identified constraints that need to be overcome for an effective relay involving any member of the Prdx family. Whether redox relays are widespread in signal transduction remains an open question and with these constraints, identification may continue to be a challenge.

## Experimental procedures

### Proteins

Human recombinant WT and C172S, C172D, and C172W Prdx2 (untagged) were prepared in the absence of DTT as described (5, 9, 32) and kindly provided by Dr Paul Pace. Reduced Prdx2 was prepared by incubation with 10 mM DTT for 1 h in pH 7.4 phosphate buffer, 0.14 M NaCl (PBS) followed by removal of the reductant using a Micro Bio-Spin 6 column (Bio-Rad). Columns were washed and pre-equilibrated with 20 mM phosphate buffer pH 7.4 containing 0.1 mM diethylenetriamine-penta-acetic acid purged with argon. Protein concentration was determined by measuring A_280_ using a NanoDrop spectrophotometer (Biolab Nanodrop Technologies) and ε_280_ = 21,430 cm^−1^M^−1^.

### Treatment of Prdx2 with GSH, SDS-PAGE, and quantification

Reduced or oxidized Prdx2 (typically 5 μM) was mixed with GSH in the presence or absence of H_2_O_2_ as stated, in 20 mM phosphate buffer pH 7.4 at 20-22 °C. Reactions were stopped at stated times for SDS-PAGE by rapid mixing with 20 mM NEM, then addition to gel loading buffer or for MS either by adding NEM or direct addition to 0.1% formic acid starting buffer.

Nonreducing SDS-PAGE was performed on 10% or 12% gels using a Bio-Rad Mini-Protean II apparatus. Gels were stained with Coomassie and visualized with an Alliance Q9 Advanced Chemiluminescence Imager (Bio-Rad). The relative intensities of the Prdx2 monomer and dimer bands were quantified by densitometry using Quantity One software (Bio-Rad).

### Stopped flow

#### Kinetics of Prdx2 glutathionylation using stopped-flow

Oxidation of Prdx2 was followed by intrinsic fluorescence changes (7, 8). Prereduced proteins (0.5 μM WT Prdx2 ∼2.5 μmol SH/μmol protein or C172S Prdx2 ∼1.5 μmol SH/μmol protein; 1 μΜ C172D or C172W Prdx2 ∼1.5 μmol SH/μmol protein) were premixed with increasing concentrations of glutathione in one syringe and the rapid change in fluorescence *(excitation ʎ*_*280nm*_*, emission above ʎ*_*320nm*_*)* was followed in a stopped-flow instrument (Applied Photophysics *SX20 MV),* after reacting with 1 or 2 μM H_2_O_2_ (2 μM H_2_O_2_ per 1 μM Prdx2). The reactions were performed at 25 °C in 50 mM sodium phosphate buffer pH 7.4 containing 100 μM diethylenetriamine-penta-acetic acid. Buffer solutions were previously treated with 10 μg/ml catalase to remove any trace of H_2_O_2_ and then filtered in an Aminco Ultra 10-kDa (Merck Millipore). An excess of glutathione was used to follow a pseudo-first order condition. Observed rate constants (*k*_obs_) for fluorescence increase were determined by fitting data to single exponential equations following the reaction up to 20 s. The values of *k*_obs_ obtained from the increasing fluorescence were plotted against glutathione concentrations and the corresponding second order rate constants were determined from the slope of these linear fittings.

### Mass spectrometry

For whole protein analysis, samples containing 1 μg protein were injected onto an Accucore-150-C4 (50 × 2.1 mm, 2.6 μm) column (60 °C) using a Dionex Ultimate 3000 HPLC system coupled to a Velos Pro mass spectrometer (Thermo Fisher Scientific) as previously (5). Proteins were eluted with an acetonitrile gradient from 90% solvent A (0.1% formic acid in water) and 10% solvent B (0.1% formic acid in acetonitrile) to 80% solvent B over 2.1 min at a flow rate of 400 μl/min. Mass spectra for all charge states were acquired between m/z 400 and 2000 in positive mode, averaged over the full-length of each protein peak and deconvoluted to yield the molecular masses and relative intensities either manually or using ProMass for Xcalibur (version 2.8 rev 5; Novatia LLC). The temperature of the capillary was 275 °C. The chromatographic conditions did not separate the different Prx species and quantification is based on relative peak intensities obtained from the deconvoluted spectral data. Analyses were performed on samples for which the reaction was stopped either by adding to 0.1% formic acid starting buffer or by adding 20 to 30 mM NEM. In the latter case, species with masses corresponding to the addition of variable numbers of NEM (mass 125) were observed and the peak intensities combined. The underivatized masses used to identify the different species are as follows: dimer, 43779; hyperoxidized dimer, 43,813; dimer-1GSH, 44,087; dimer-2GSH, 44,392; monomer, 21,892; monomer-1GSH, 22,197; monomer-2GSH, 22,502. The accuracy of the deconvoluted masses was within 5 Da of theoretical masses and peaks corresponding to <5% of the total signal were not quantified. Percentages are based on signal intensity.

### Data analysis

To estimate kinetic parameters, the time courses of dimer and monomer fractions computed from the densitometry of SDS-PAGE gels in experiments following Prdx2 glutathionylation were fitted by suitable kinetic models. Numerical simulations and fits were carried out in *Mathematica* v.14.0.0.0 [Wolfram Research Inc. (2024) *Mathematica*, Version 14, Wolfram Research, Inc]. Modeling assumptions and details of the analyses are presented in SI3.

## Data availability

All data are contained within the manuscript.

## Supporting information

This article contains supporting information (5, 9, 33, 34, 35, 36).

## Conflict of interest

The authors declare that they have no conflicts of interest with the contents of this article.

## Supporting information

Peskin Supporting Information

## Footnotes

1 We previously interpreted a decrease in the staining of Western blots with antiPrdxSO2/3 antibody as evidence for protection by GSH against hyperoxidation (ref 5) but we have subsequently found that this decrease can be explained by the antibody reacting better with the hyperoxidized dimer than hyperoxidized monomer.
