# Supplementary material for: Mechanism of glutathionylation of the active site thiols of peroxiredoxin 2
Source: J Biol Chem. 2025 Apr 11;301(5):108503. doi: 10.1016/j.jbc.2025.108503 (PMC12137162; doi:10.1016/j.jbc.2025.108503)
Supplement: Peskin Supporting Information [file mmc1.pdf]

Supporting Information for

Mechanism of glutathionylation of the active site thiols of peroxiredoxin 2

Alexander V. Peskin[1], Flavia C. Meotti[2], Nicholas J. Magon[1], Luiz P. de Souza[2], Armindo Salvador[3,4,5,6*] and Christine C. Winterbourn[1*]

[1] Mātai Hāora - Centre for Redox Biology and Medicine, Department of Pathology and Biomedical Science, University of Otago Christchurch, New Zealand; [2] Department of Biochemistry, Chemistry Institute, University of Sao Paulo, Sao Paulo-SP, Brazil; [3] CNC-UC - Centre for Neuroscience Cell Biology, University of Coimbra; [4] CiBB - Centre for Innovative Biomedicine and Biotechnology, University of Coimbra; [5] Coimbra Chemistry Center - Institute of Molecular Sciences (CQC-IMS), University of Coimbra; [6] Institute for Interdisciplinary Research, University of Coimbra, Coimbra, Portugal

Table of Contents

1 Symbols list 2

2 Figures from experiments 4

3 Estimation of the rate and equilibrium constants for thiol-disulfide exchange between Prdx2-SS and GSH 9

3.1 Thiol-disulfide exchange between double-disulfide dimers and GSH 9

3.2 Thiol-disulfide exchange between GSH and the disulfide in partially glutathionylated dimers, and potential influence of glutathionylation at one site on glutathionylation of the other 19

3.2.1 Analysis of the experiments in the absence of H_2O_2 19

3.2.2 Analysis of the experiments in the presence of H_2O_2 23

4 References 29

1 Symbols list

Symbol	Meaning
c_f	Pre-exponential coefficient of the fast component in bi-exponential decay
c_s	Pre-exponential coefficient of the slow component in bi-exponential decay
δ	Adjustable parameter interpreted as $k_{GP,SS} + k_{GR,SS}$
$\delta_{H_2O_2}$	0 if H_2O_2 is absent and 1 otherwise
f_0	Fraction of Prdx2 dimeric units with no disulfide bonds
f_1	Fraction of Prdx2 dimeric units with one disulfide bond
f_{1P}	Fraction of C_P-monoglutathionylated, disulfide crosslinked dimers
f_2	Fraction of Prdx2 dimeric units with two disulfide bonds
$f_{2,0}$	Initial fraction of Prdx2 dimeric units with two disulfide bonds
$f_{2,\infty}$	Final fraction of Prdx2 dimeric units with two disulfide bonds
f_R	Total fraction of glutathionylated Prdx2 active sites
γ	Adjustable parameter interpreted as $k_{-GP,SS} + k_{TPR,SS}$
$k_{D,x}$	Rate constant for GSH-dependent deglutathionylation when the other active site in the same dimer is in state *x*, with *x*= SH, SS, SSG or SSGSSG for thiol, disulfide, monoglutathionylated or diglutathionylated, respectively. Absent *x* means that the rate constant is assumed to be independent of the other site's state.
k_f	Fast characteristic constant of a biexponential decay
$k_{G,x}$	Rate constant for thiol-disulfide exchange between GSH and Prdx2 disulfide yielding any (C_P- or C_R-glutathionylated) product when the other active site in the same dimeric unit is in state *x*.
$k_{-G,x}$	Rate constant for self-deglutathionylation of Prdx2 monoglutathionylated active site when the other site in the same dimeric unit is in state *x*.
$k_{GP,x}$	Rate constant for thiol-disulfide exchange between GSH and Prdx2 disulfide yielding a C_P-glutathionylated product when the other active site in the same dimeric unit is in state *x*.
$k_{-GP,x}$	Rate constant for self-deglutathionylation from C_P when the other active site in the same dimeric unit is in state *x*.
$K_{-GP,x}$	Equilibrium constant for self-deglutathionylation from C_P when the other active site in the same dimeric unit is in state *x*.
$k_{-GPR,x}$	Rate constant for self-deglutathionylation from either C_P or C_R when the other active site in the same dimeric unit is in state *x*.
$k_{GR,x}$	Rate constant for thiol-disulfide exchange between GSH and Prdx2 yielding a C_R-glutathionylated product when the other active site in the same dimeric unit is in state *x*.
$k_{-GR,x}$	Rate constant for self-deglutathionylation from C_R when the other active site in the same dimeric unit is in state *x*.
$K_{-G,x}$	Equilibrium constant for self-deglutathionylation of a Prdx2 active site when the other site in the same dimeric unit is in state *x*.
$K_{-GR,x}$	Equilibrium constant for self-deglutathionylation from C_P when the other active site in the same dimeric unit is in state *x*.
k_s	Slow characteristic constant of a biexponential decay

$k_{TPR,x}$	Rate constant for transfer of the glutathionyl moiety from C_P to C_R when the other active site in the same dimeric unit is in state x.
$k_{TRP,x}$	Rate constant for transfer of the glutathionyl moiety from C_R to C_P when the other active site in the same dimeric unit is in state x.
$K_{TRP,x}$	Equilibrium constant for transfer of the glutathionyl moiety from C_R to C_P when the other active site in the same dimeric unit is in state x.
$Prdx_{\mathrm{Tot}}$	Total concentration of Prdx2 monomers.
θ	Adjustable parameter interpreted as $k_{GP,SS} k_{-GP,SS}$
$r_{x,y}$	Ratio between the rate constant for reaction x when the other active site in the same dimeric unit is in state y and the rate constant for the same reaction when the other active site is in disulfide form.
$R_{x,y}$	Ratio between the equilibrium constant for reaction x when the other active site in the same dimeric unit is in state y and the equilibrium constant for the same reaction when the other active site is in disulfide form.
t	Time

2 Figures from experiments

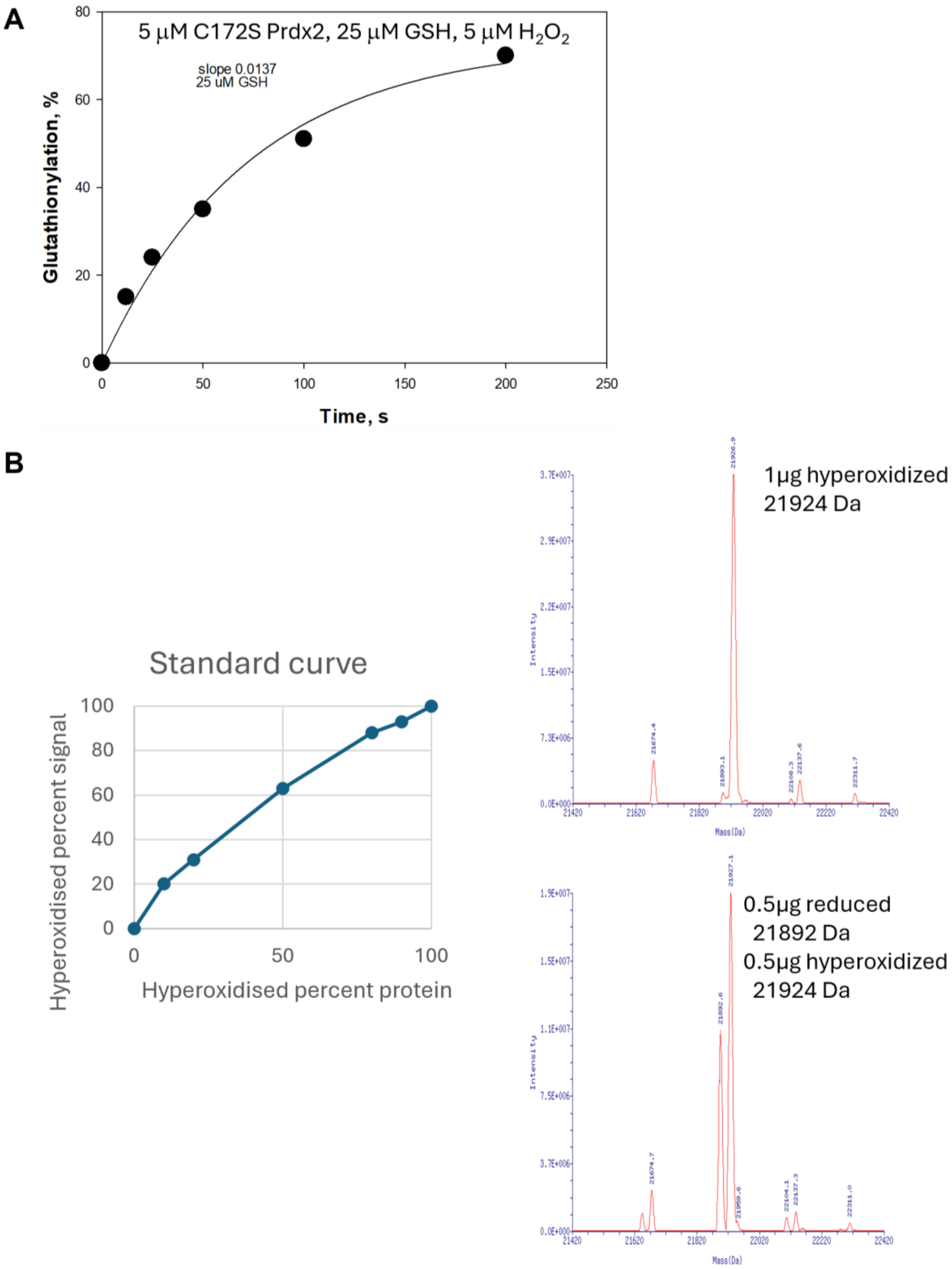

Figure S1. (A) Time course of glutathionylation of reduced Prdx2 C172S mutant (5 μM) treated with equimolar H_2O_2 in the presence of 25 μM GSH. *Reaction was stopped at stated times by adding NEM (10 mM) and left on the bench for 1h before LC/MS. Treated protein in the absence of GSH gave the derivatised sulfenic acid (M+16+125) and progressive increase with [GSH] in glutathionylated product (M+306). Quantification is based on signal intensity.* ***(B) Standard curve for quantification of reduced and hyperoxidized Prdx2.*** *WT Prdx2 was reduced with 10 mM DTT or treated with 10 mM DTT and 5 mM H_2O_2 to produce the sulfinic acid. Reduced and hyperoxidized proteins were passed through a MicroBio-Spin 6 columns (Bio-Rad) separately. These were mixed in stated proportions and analyzed by whole protein MS. Data show a greater response to the hyperoxidized compared with reduced protein.*

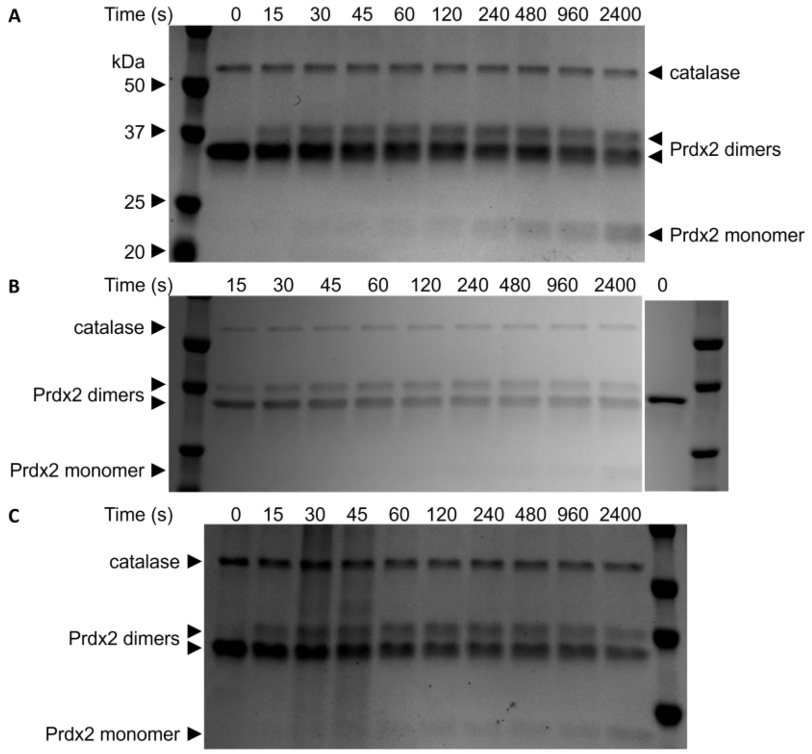

Figure S2. Time course of Prdx2 disulfide reduction upon incubation of 5 µM Prdx2-SS with 4 mM GSH in the presence of 10-20 µg/ml catalase, terminated by 20-30 mM NEM. *A, B & C show Coomassie stained non-reducing SDS-PAGE gels for 3 independent experiments. The molecular weight markers are indicated to the left of the gel in A. They are the same for all the gels shown in this work. The gel in panel B is reproduced from Figure 3B.*

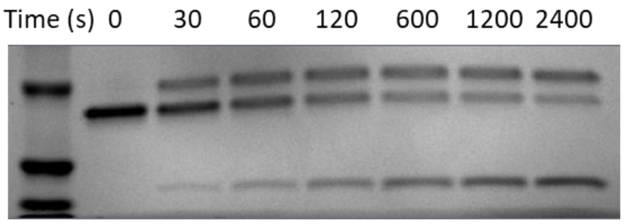

Densitometry %						
1DS	32	38	40	42	40	42
2DS	62	46	35	27	23	18
M	6	16	25	31	37	40

Figure S3. Time course of disulfide exchange between oxidized Prdx2 (5 µM) and GSH (10 mM) in the absence of catalase. *This shows an enhanced reaction in the absence of catalase (cf. Figure S2): For comparison, the 16% conversion to monomer at 1 min and 40 % at 40 min relate to means (SD) of 3 (3) % and 19 (6) % respectively under similar conditions with catalase present (Fig. S2). M, monomer; DS, disulfide.*

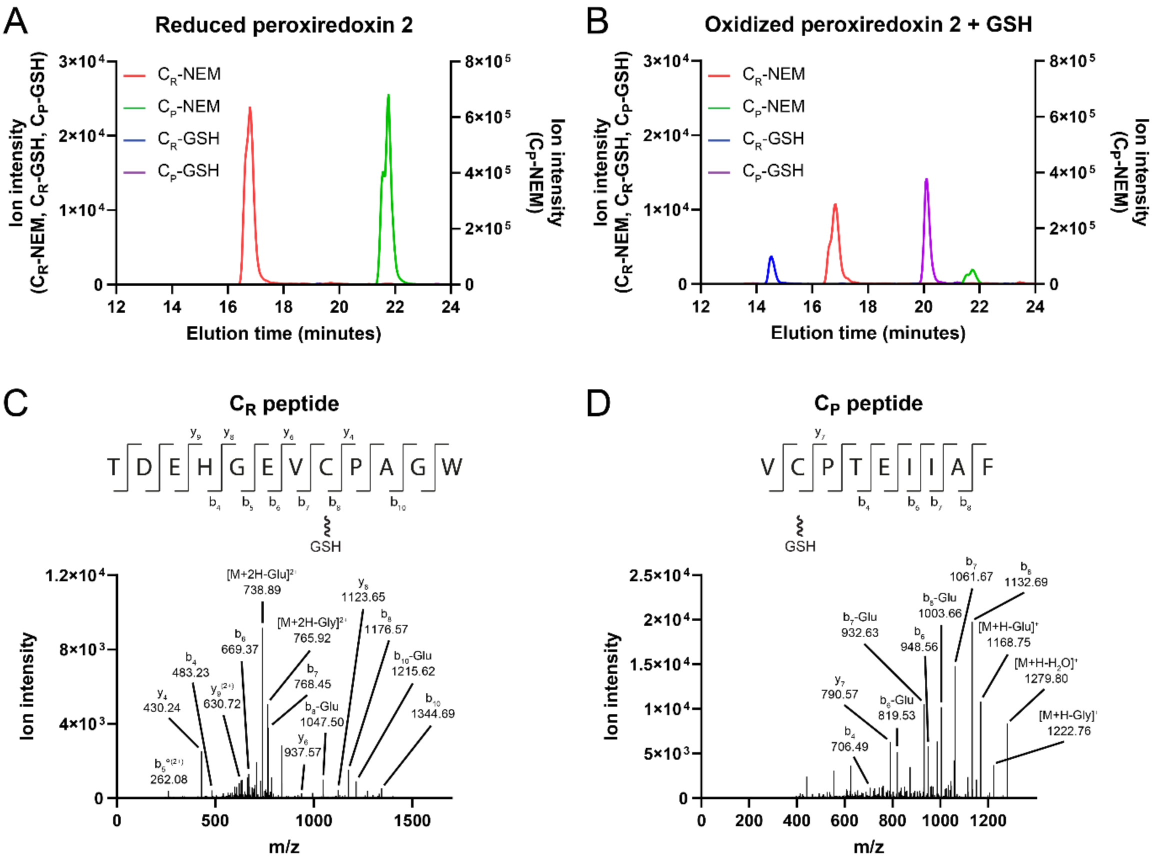

Figure S4. Glutathionylation of Prdx2 C_P and C_R following incubation of oxidized Prdx2 with GSH. *Selected MS/MS ion chromatograms for chymotryptic peptides of (**A**) reduced Prdx2 control and (**B**) oxidized Prdx2 after 15 s treatment with 4 mM GSH. Samples were treated with 20 mM NEM to block reduced cysteine residues. Fragmentation patterns of (**C**) glutathionylated C_R peptide (803.30 m/z, 14.5 minutes), and (**D**) glutathionylated C_P peptide (1297.58 m/z, 20.1 minutes). Samples were digested at a 20:1 substrate:chymotrypsin weight ratio at 25 °C overnight then analyzed using a Thermo Scientific Velos Pro ion trap mass spectrometer coupled to a Dionex UltiMate 3000 HPLC system with a 50 µL injection loop (Thermo Scientific, Waltham, MA, USA). A Jupiter 4-µm Proteo 90Å column (150 x 2 mm, Phenomenex, Torrance, CA) was used for chromatographic separation using a water/acetonitrile (0.1% formic acid) gradient as described (Peskin et al. J Biol Chem 291, 3053-62, 2016). Data were analyzed using Thermo Xcalibur Qual Browser 4.2.47 (Thermo Fisher Scientific Inc., Waltham, MA, USA). The m/z values of peptides of interest were predicted and collision-induced dissociation-MS/MS spectra in positive-ion mode were acquired for each. Peptide fragments were manually assigned based on Roepstorff-Fohlman nomenclature. Chromatograms for each peptide species were obtained by post-acquisition filtering the MS/MS spectra obtained for a chosen abundant and characteristic fragment ion. C_R-NEM (parent: 713.29 m/z, fragment: 996.37 m/z), C_P-NEM (parent: 1117.56 m/z, fragment: 881.44 m/z), C_R-GSH (parent: 803.30 m/z, fragment: 765.79 m/z), C_R-GSH (parent: 1297.58 m/z, fragment: 1132.50 m/z).*

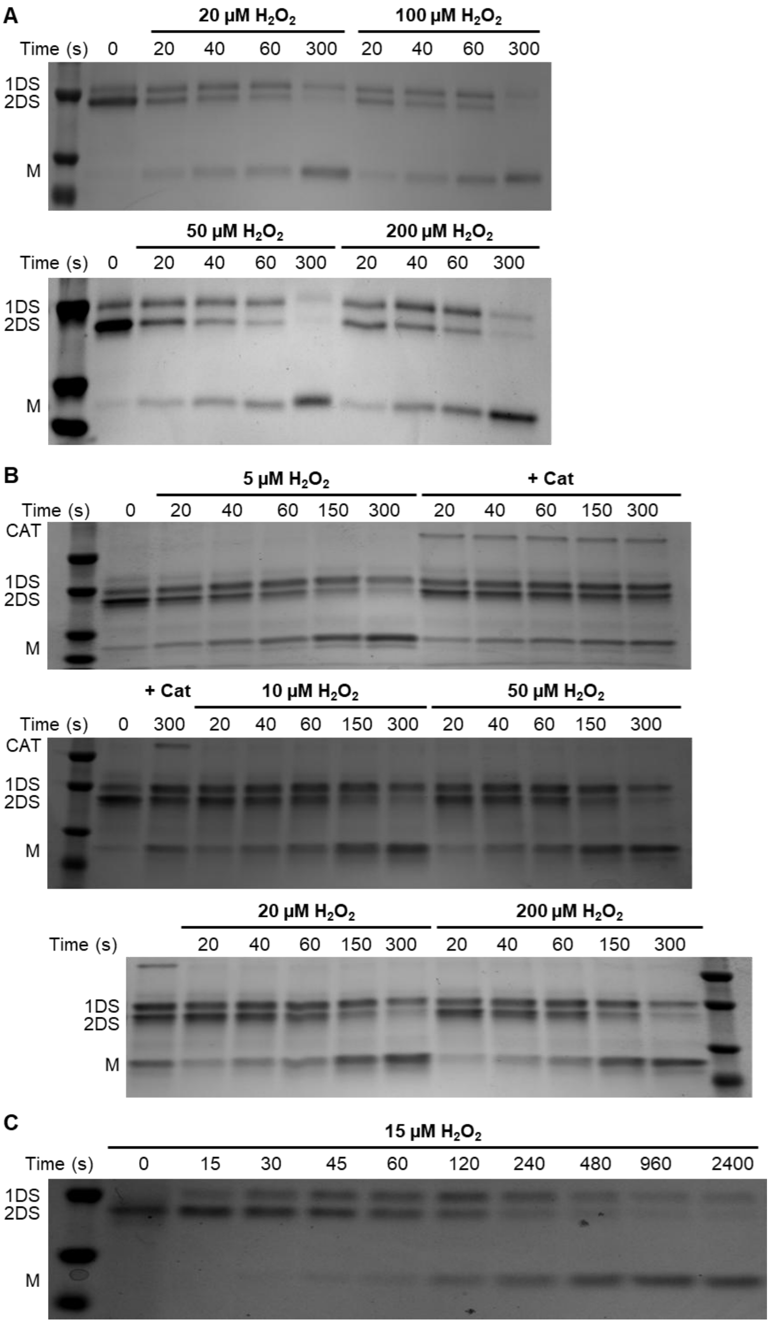

Figure S5. Time course of glutathionylation of 5 μM oxidized Prdx2 treated with H_2O_2 and 8 mM (A, B) or 4 mM (C) GSH. *Non-reducing SDS-PAGE of changes in Prdx2 monomers and dimers over time for the indicated H_2O_2 concentrations from three independent experiments. Reaction was stopped at stated times with 20-30 mM NEM. Densitometry results are plotted in Figure S9A,B,D, respectively.*

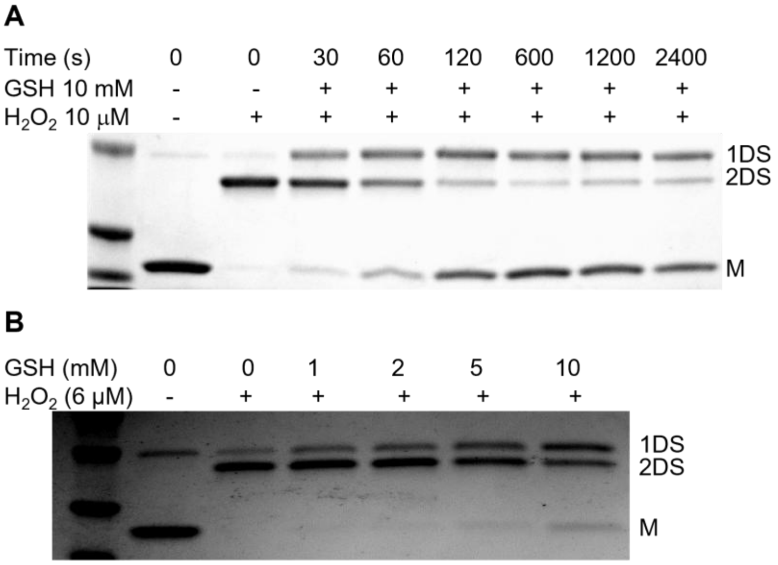

Densitometry (%)						
**1DS**	17	16	24	26	37	44
**2DS**	0	84	76	71	56	39
**M**	83	0	0	3	7	17

Figure S6. Glutathionylation of reduced Prdx2 (5 µM) treated with GSH and H_2O_2: *Supporting information for Figure 4C showing results from additional experiments carried out under comparable conditions. (**A**) Non-reducing SDS-PAGE gel showing time dependent changes following treatment of 5 µM Prdx2 with 10 mM GSH and 10 µM H_2O_2. (**B**) Gel showing short term (10 s) changes on treating 5 µM Prdx2 with 6 µM H_2O_2 and varying concentrations of GSH.*

3 Estimation of the rate and equilibrium constants for thiol-disulfide exchange between Prdx2-SS and GSH

3.1 Thiol-disulfide exchange between double-disulfide dimers and GSH

In non-reducing SDS-PAGE gels, dimeric units that are not cross-linked by any disulfide run as monomers, and doubly cross-linked (2DS) dimers run slightly faster than singly cross-linked (1DS) ones. In the present case, the relative densities of these bands reflect the progressive glutathionylation of 2DS dimers in the presence of GSH in the following characteristic way (Figure S2). The gradual attenuation of the 2DS band is accompanied by the densification of a 1DS band. This is already evident 15 s after GSH addition, corresponding to dimers that are glutathionylated at a single site. This band subsequently further densifies, and by 30 s – 60 s minutes a monomer band becomes detectable, which also subsequently densifies as the 1DS band slightly fades. As substantiated by MS studies (see Figure S4), this early-appearing monomer band ensues mainly from monoglutathionylation of one of the disulfides. An excess of catalase was added at the beginning in order to avoid artifactual Prdx2 oxidation by adventitious H_2O_2, as evident in Figure S3. The proportions of the three Prdx2 bands are determined by densitometry.

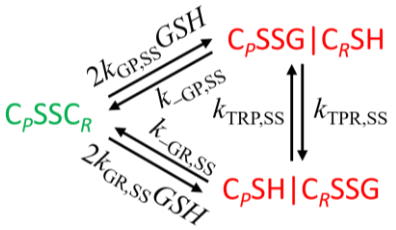

Figure S7. Reaction scheme underlying the model for thiol-disulfide exchange between GSH and a Prdx2 disulfide at a single active site. *The species represented by green and red symbols run as 2DS and 1DS dimers in non-reducing SDS-PAGE gels, respectively. The (disulfide) state of the second active site in the dimer is omitted for simplicity.*

We will begin by analyzing the decay of the fraction of 2DS dimeric units over the first 4 min. As little monomer accumulates over this initial period, analysis can be based on a simple single-active-site model. The model must take the following considerations into account. (i) The reaction of GSH with 2DS dimers can generate two distinct glutathionylated products, one glutathionylated at C_P and the other at C_R; (ii) the glutathionyl moiety may exchange between these two Cys in an active site; (iii) there is significant formation of *both* the C_P- and the C_R-glutathionylated products already by 15 s after GSH addition (Figure S4). (iv) The relative amounts of disulfide (2DS) and mono-glutathionylated (1DS) products equilibrate within the 4 min time window (Figure S2). These considerations lead to the reaction scheme in Figure S7. Considering additionally that GSH is present in a pseudo-first-order excess over Prdx2, this reaction scheme translates into the equations below:

$$\begin{aligned}
\frac{dC_PSSC_R}{dt} &= k_{-GP,SS}\times C_PSSG\,|\,C_RSH + k_{-GR,SS}\times C_PSH\,|\,C_RSSG - \\
&\quad - 2\times(k_{GP,SS}+k_{GR,SS})\times C_PSSC_R\times GSH \\
\frac{dC_PSSG\,|\,C_RSH}{dt} &= 2\times k_{GP,SS}\times C_PSSC_R\times GSH + k_{TRP,SS}\times C_PSH\,|\,C_RSSG - \\
&\quad - (k_{-GP,SS}+k_{TPR,SS})\times C_PSSG\,|\,C_RSH \\
C_PSSC_R &+ C_PSSG\,|\,C_RSH + C_PSH\,|\,C_RSSG = \frac{1}{2}Prdx2_{\mathrm{Tot}},
\end{aligned} \tag{1}$$

where $Prdx2_{\mathrm{Tot}}$ stands for the total concentration of Prdx2 monomers and the rate constants are as indicated in Figure S7. Solving this system and rearranging yields a solution for the decay of the fraction of 2DS dimeric units in the form:

$$f_2(t) = f_{2,\infty}\left(1 + c_f e^{-k_f t} + c_s e^{-k_s t}\right). \tag{2}$$

Here,

$$f_{2,\infty} = \frac{f_{2,0}}{1 + 2\times\left(\frac{k_{GP,SS}}{k_{-GP,SS}} + \frac{k_{GR,SS}}{k_{-GR,SS}}\right)\times GSH}, \tag{3}$$

with $f_{2,0}$ the initial fraction of 2DS dimeric units, stands for the (quasi-)equilibrium fraction of 2DS dimeric units,

$$c_f = GSH\left(\frac{k_{GP,SS}}{k_{-GP,SS}} + \frac{k_{GR,SS}}{k_{-GR,SS}} + R\right), \quad c_s = GSH\left(\frac{k_{GP,SS}}{k_{-GP,SS}} + \frac{k_{GR,SS}}{k_{-GR,SS}} - R\right), \tag{4}$$

with

$$R = \frac{k_{GR,SS}\left(1 - \frac{k_{-GP,SS}}{k_{-GR,SS}}\right)\left(1 - \frac{k_{TRP,SS}}{k_{TPR,SS}}\right) + \left(2GSH(k_{GP,SS} + k_{GR,SS}) - k_{TPR,SS} - k_{TRP,SS}\right)\frac{k_{GR,SS}}{k_{-GR,SS}}\left(1 + \frac{k_{TRP,SS}}{k_{TPR,SS}}\right)}{\sqrt{\begin{array}{l}\left(2GSH(k_{GP,SS} + k_{GR,SS}) + k_{-GP,SS} + k_{-GR,SS} + k_{TPR,SS} + k_{TRP,SS}\right)^2 - \\ \quad -4\left(\frac{k_{-GR,SS}}{k_{GR,SS}} + 2GSH\left(1 + \frac{k_{TRP,SS}}{k_{TPR,SS}}\right)\right)\left((k_{GP,SS} + k_{GR,SS})k_{TPR,SS} + k_{GR,SS}k_{-GP,SS}\right)\end{array}}}$$

(5)

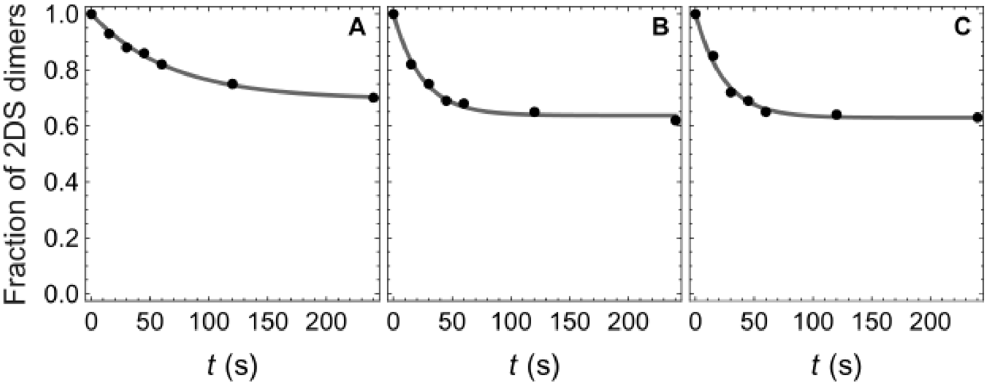

	A	B	C	Mean±SEM
$k_{G,SS} \doteq k_{GP,SS}+k_{GR,SS}$ ($M^{-1}s^{-1}$)	0.570 ± 0.025	1.87 ± 0.12	1.91 ± 0.13	1.45 ± 0.06
$k_{-GPR,SS} \doteq k_{-GP,SS}=k_{-GR,SS}$ (s^{-1})	0.0104 ± 0.0008	0.0262 ± 0.0023	0.0259 ± 0.0025	0.0208 ± 0.0012
Adj. R^2	0.99992	0.9997	0.9997	–

Figure S8. Time course of the decay of the fraction of 2DS dimers over the first 4 minutes of the experiments shown in Figure S2. *The lines show the fits of the mono-exponential equation* (21)*, to the data. The table shows the best-fit parameter estimates obtained from these fits and the goodness-of-fit statistics. Fits were done in Mathematica™ v14.0.0.0 using the function NonlinearModelFit with the option MaxIterations → 1000.*

are the pre-exponential coefficients for the fast and slow components, respectively, and

$$k_f = \frac{1}{2}\left(2GSH\,(k_{GP,SS} + k_{GR,SS}) + k_{-GP,SS} + k_{-GR,SS} + k_{TPR,SS} + k_{TRP,SS} + A\right),$$
$$k_s = \frac{1}{2}\left(2GSH\,(k_{GP,SS} + k_{GR,SS}) + k_{-GP,SS} + k_{-GR,SS} + k_{TPR,SS} + k_{TRP,SS} - A\right), \tag{6}$$

with

$$A = \sqrt{\begin{array}{l}\left(2GSH\,(k_{GP,SS} + k_{GR,SS}) + k_{-GP,SS} + k_{-GR,SS} + k_{TPR,SS} + k_{TRP,SS}\right)^2 - \\ -4\left(\begin{array}{l}2GSH\left(k_{GR,SS}k_{-GP,SS} + k_{GP,SS}k_{-GR,SS} + (k_{GP,SS} + k_{GR,SS})(k_{TPR,SS} + k_{TRP,SS})\right) \\ +k_{-GR,SS}k_{TPR,SS} + k_{-GP,SS}(k_{-GR,SS} + k_{TRP,SS})\end{array}\right)\end{array}}$$

(7)

are the characteristic constants for the fast and slow components, respectively. The six rate constants are linked through the following micro-reversibility constraint:

$$\frac{k_{-GR,SS}k_{GP,SS}k_{TPR,SS}}{k_{GR,SS}k_{-GP,SS}k_{TRP,SS}} = 1 \tag{8}$$

Note that this model, which we will henceforth denote by **Model 1**, predicts a bi-exponential decay even if no significant transfer of the glutathionyl (GS) moiety between C_P and C_R occurs. However, the observed decay is strictly mono-exponential over the first 4 min, according to the fits shown in Figure S8, which also presents the obtained parameter estimates. The lack of one of the expected dynamic components of the decay might be due to very fast equilibration of one of the three thiol-disulfide exchange reactions, or to $k_{-GP,SS}$ and $k_{-GR,SS}$ having nearly identical values.

To investigate what is the correct explanation, and to achieve a finer-grained understanding of the kinetics of glutathionylation, we now examine the decay of the fraction of 2DS dimeric units in the presence of both GSH and H_2O_2 under a range of conditions such as shown as gels in Figures S5 and 4B and with the densitometry quantified in Figure S9A-D. The presence of H_2O_2 consistently increased the rate and extent of decrease of 2DS dimers (Figures S9B, and 4A&B from the main text), suggesting that a reaction of H_2O_2 with, presumably, the C_P-SH of the C_R-glutathionylated product displaces the Prdx2-SS – GSH thiol-disulfide exchange equilibrium. The conjecture that this thiol retains H_2O_2 reactivity is consistent with the observation that mutation of the resolving Cys to a Trp, which strongly distorts the active site, still allows H_2O_2 to oxidize the C_P-SH with a $(1.35 \pm 0.05)\times10^6$ $M^{-1}s^{-1}$ rate constant (Peskin et al., 2021). Moreover, the formation of a C_PSOH|C_RSSG oxidation product is consistent with the formation of diglutathionylated species by subsequent condensation with GSH, as seen in Figure 5A&B. The effect of H_2O_2 on the decrease of 2DS dimers is already fully saturated at H_2O_2 concentrations above ≈20 μM,[1] indicating that at these high concentrations the processes

[1] This is a conservative value, supported by two independent experiments (Figure S9A&B). However, saturation may occur at even lower H_2O_2 concentrations. Thus, in the experiment in Figure S9B the best fit estimates for 5 and 10 μM H_2O_2 do not significantly differ from those obtained for ≥20 μM H_2O_2 (Figure S9F). And the independent experiment in Figure S9D, using 15 μM H_2O_2, yielded best-fit estimates (Table S2) in the same range as those obtained for ≥20 μM H_2O_2 in the experiments in Figure S9A&B.

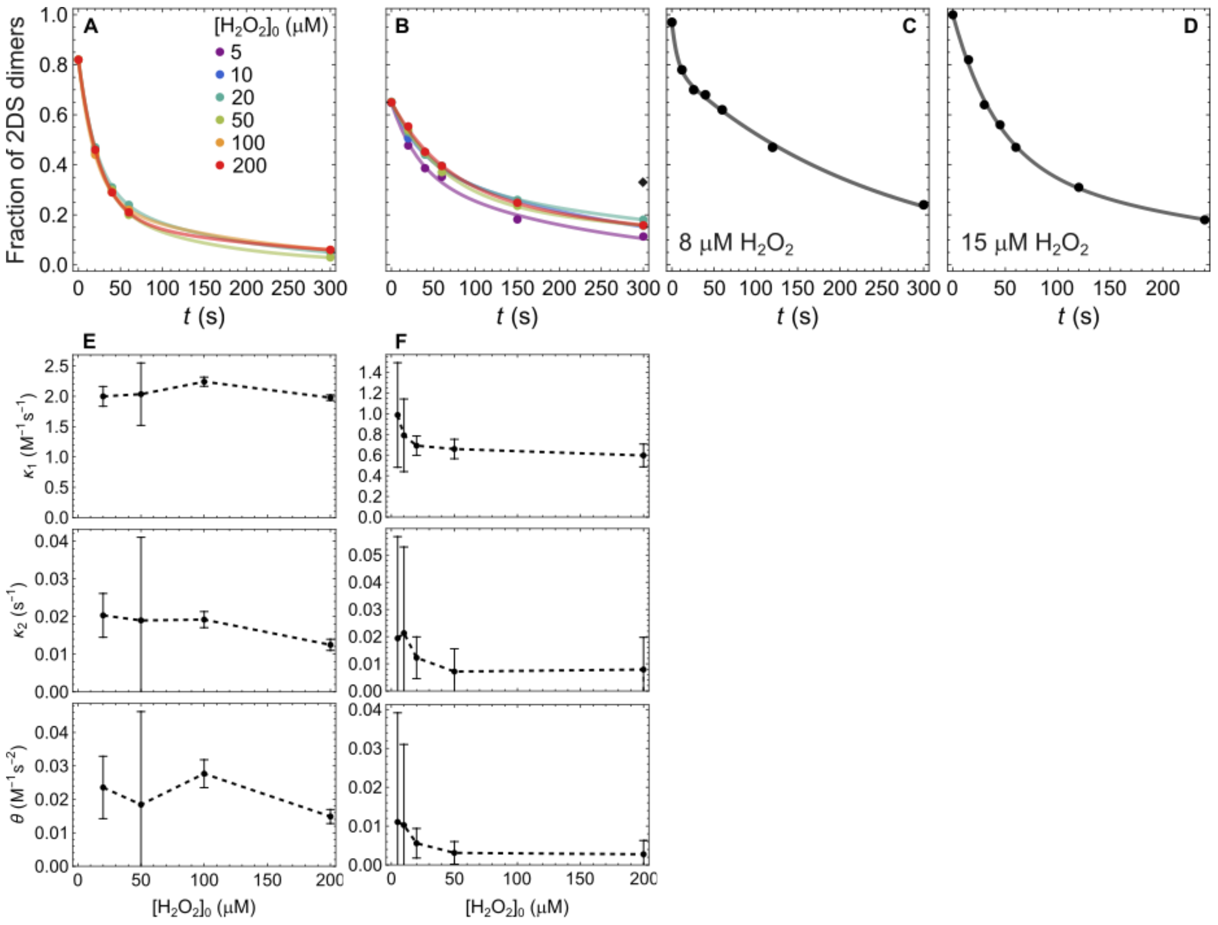

Figure S9. Fits to the time courses of the fraction of 2DS dimers upon incubation of 5 μM Prdx2-SS with 8 mM (A,B), 5 mM (C) or 4 mM (D) GSH. *Gel data for A,B and D are from Figure S5A,B and C, respectively, with the legend showing indicated H_2O_2 concentration; gel data for C from Figure 4B. (E,F) Parameter estimates obtained by fitting Model 2 to the data sets in A and B, respectively. The estimates and bounds obtained from the data in A and B are shown in Table S1, the average values from these experiments and the values obtained from the data in C and D are shown in Table S2. The adjusted R^2 values for the fits shown in C, D are 0.9997 and 0.9995, respectively. The dots represent experimental determinations, the lines show the fits of bi-exponential Model 2 to the time courses for each initial H_2O_2 concentration. The black diamond in (B) indicates the fraction of 2DS dimer by 300 s for an incubation in the presence of catalase. In panels E and F, the error bars represent 95% confidence intervals, the dashed lines are guides to the eye. Fits were done in Mathematica™ v14.0.0.0 using the function NonlinearModelFit with default options.*

releasing the C_P-SH for oxidation become rate-limiting (Figure S5). This is the situation modelled below.

Release of the C_P-SH for oxidation may occur immediately upon attack of GSH on the Prdx2 disulfide forming the C_RSSG adduct as a primary product, or (non-exclusively) after $C_P \rightarrow C_R$ transfer of the adduct from a primary C_P-SSG product. These considerations lead to the simple reaction scheme in Figure S10 for the early reactions of a single Prdx2-SS site upon incubation with GSH and kinetically saturating H_2O_2, which translates into the following kinetic model for the evolution of the fraction (f_2) of 2DS dimeric units:

$$\begin{aligned} \frac{df_2}{dt} &= k_{-\mathrm{GP,SS}}\, f_{1\mathrm{P}} - 2(k_{\mathrm{GP,SS}} + k_{\mathrm{GR,SS}})\, GSH\ f_2 \\ \frac{df_{1\mathrm{P}}}{dt} &= 2k_{\mathrm{GP,SS}}\, GSH\ f_2 - (k_{-\mathrm{GP,SS}} + k_{\mathrm{TPR,SS}})\, f_{1\mathrm{P}}. \end{aligned} \quad (9)$$

Here, f_{1P} stands for the fraction of dimeric units in C*P*SSG|C*R*SH•C*P*SSC*R* form. Considering the concentration of GSH fixed, the solution of this system for f_2 can be written as:

$$f_2(t)=\frac{1}{2}f_2(0)\left(\left(1+\frac{2\gamma GSH-\delta}{\sqrt{(2\gamma GSH-\delta)^2-8\theta GSH}}\right)e^{-\frac{1}{2}\left(2\gamma GSH+\delta+\sqrt{(2\gamma GSH-\delta)^2-8\theta GSH}\right)t}+\right.$$
$$\left.+\left(1-\frac{2\gamma GSH-\delta}{\sqrt{(2\gamma GSH-\delta)^2-8\theta GSH}}\right)e^{-\frac{1}{2}\left(2\gamma GSH+\delta-\sqrt{(2\gamma GSH-\delta)^2-8\theta GSH}\right)t}\right), \tag{10}$$

with:

$$\gamma=k_{\text{GP,SS}}+k_{\text{GR,SS}}, \tag{11}$$

$$\delta=k_{-\text{GP,SS}}+k_{\text{TPR,SS}}, \tag{12}$$

$$\theta=k_{\text{GP,SS}}k_{-\text{GP,SS}}. \tag{13}$$

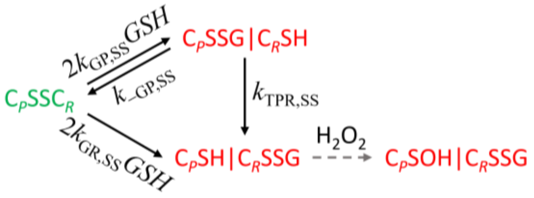

Figure S10. Reaction scheme underlying the model to fit the decay of 2DS Prdx2 dimers in the presence of GSH and kinetically saturating H_2O_2 concentrations. *Green and red symbols indicate 2DS and 1DS dimers, respectively. The model does not explicitly consider the reaction in dashed gray, which is shown just to indicate that glutathionylation at C_P and $C_P \rightarrow C_R$ GS transfer are considered irreversible due to the strong competition of sulfenylation with the respective reverse reactions. The (disulfide) state of the second active site in the dimer is omitted for simplicity.*

Therefore, the individual mechanistic rate constants are not identifiable. What the fits of this model — henceforth denoted by **Model 2** — to the decay of f_2 allow to estimate are the aggregated parameters γ, δ and θ, which we denoted by Greek letters to keep the distinction clear in the discussion that follows. Of these, γ and δ can be interpreted as the overall rate constants for glutathionylation and for removal of the GS moiety from C*P*, respectively. Rearranging expressions (11), (12), (13), we find that the estimates for γ, δ and θ impose the following constraints between the mechanistic rate constants:

$$k_{GP,SS}=\gamma-k_{GR,SS}, \tag{14}$$

$$k_{-GP,SS}=\frac{\theta}{\gamma-k_{GR,SS}}, \tag{15}$$

$$k_{TPR,SS}=\delta-\frac{\theta}{\gamma-k_{GR,SS}}. \tag{16}$$

Of note, the upper bound for $k_{GR,SS}$ corresponds to a 0 value for $k_{TPR,SS}$ and lower and upper bounds for $k_{GP,SS}$ and $k_{-GP,SS}$, respectively. These and other bounds for the mechanistic rate and equilibrium constants can be computed through suitable analysis of equations (8), (11), (12), (13):

$$\frac{\theta}{\delta}<k_{GP,SS}<\gamma \tag{17}$$

$$\frac{\theta}{\gamma} < k_{-GP,SS} < \delta \tag{18}$$

$$\frac{\theta}{\gamma^2} < K_{-GP,SS} < \frac{\delta^2}{\theta} \tag{19}$$

$$k_{GR,SS} < \gamma - \frac{\theta}{\delta} \tag{20}$$

$$\frac{k_{GP,SS}}{k_{GR,SS}} > \frac{\theta}{\delta\gamma - \theta} \tag{21}$$

$$k_{TPR,SS} < \delta - \frac{\theta}{\gamma} \tag{22}$$

$$\frac{k_{TPR,SS}}{k_{-GP,SS}} < \frac{\delta\gamma}{\theta} - 1 \tag{23}$$

The values obtained from the fits are shown in Table S1. The model also excellently fitted the decay of f_2 in additional experiments carried out using 5 mM GSH + 8 µM H_2O_2 (Figure S9C) or 4 mM GSH + 15 µM H_2O_2 (Figure S9D). The latter experiment yielded best-fit estimates in the same range, whereas the former yielded somewhat higher estimates of all the aggregated parameters. The estimates obtained from these experiments are summarized in Table S2.

The results show that none of the three reversible processes in Figure S7 could have equilibrated within the 15 s that mediate to the first experimental time point, for the following two reasons. First, the transfer of the GS moiety from C_P to C_R and deglutathionylation from C_P are both relatively slow. Second, as glutathionylation at C_R is slower than glutathionylation at C_P, if deglutathionylation from C_R were as fast as to allow equilibration within 15 s very little C_R-glutathionylated product would form, contrary to observations. Therefore, only one explanation for the mono-exponential decay of the fraction of 2DS dimeric units in the experiments where Prdx2-SS was incubated with GSH in the absence of H_2O_2 is consistent with all the data. Namely, that the rate constants for deglutathionylation from C_P and from C_R are similar $(k_{-GR,SS} \approx k_{-GP,SS})$. Under these conditions, Equation (2) simplifies to

$$f_2(t) = f_2(0)\frac{k_{-GPR,SS} + 2k_{G,SS}GSH\ e^{-(k_{-GPR,SS}+2k_{G,SS}GSH)t}}{k_{-GPR,SS} + 2k_{G,SS}GSH}, \tag{24}$$

where $k_{G,SS} \doteq k_{GR,SS} + k_{GP,SS} (= \gamma)$ and $k_{-GPR,SS} \doteq k_{-GR,SS} = k_{-GP,SS}$. Moreover, the conjecture that $k_{-GR,SS} \approx k_{-GP,SS}$ yields the following additional bounds:

$$K_{-GR,SS} > \begin{cases} \dfrac{\delta^2}{\delta\gamma - \theta} \Leftarrow \delta\gamma \le 2\theta \\ \dfrac{4\theta}{\gamma^2} \Leftarrow \delta\gamma > 2\theta \end{cases} \tag{25}$$

Table S1. Best-fit estimates and bounds obtained from the data shown in Figure S9A, B (tables A and B, respectively), with respective standard errors. *The last column shows variance-weighted means and standard errors of the best-fit estimates and bounds obtained for each of the runs with [H_2O_2] ≥ 20 μM.*

**A**	**20 μM H_2O_2**	**50 μM H_2O_2**	**100 μM H_2O_2**	**200 μM H_2O_2**	**Mean**
$\gamma = k_{GR,SS} + k_{GP,SS}$ ($M^{-1}s^{-1}$)	2.00 ±0.04	2.03 ±0.12	2.240 ±0.018	1.977 ±0.011	2.046 ±0.009
$\delta = k_{-GP,SS} + k_{TPR,SS}$ (s^{-1})	0.0203 ±0.0014	0.019 ±0.005	0.0192 ±0.0005	0.01248 ±0.00034	0.01491 ±0.00028
$\theta = k_{GP,SS} k_{-GP,SS}$ ($M^{-1}s^{-2}$)	0.0236 ±0.0022	0.018 ±0.006	0.0277 ±0.0010	0.0149 ±0.0005	0.0177 ±0.0004
$k_{GP,SS,min}$ ($M^{-1}s^{-1}$)	1.16 ±0.13	1.0 ±0.4	1.44 ±0.06	1.19 ±0.05	1.28 ±0.04
$k_{GP,SS,max}$ ($M^{-1}s^{-1}$)	2.00 ±0.04	2.03 ±0.12	2.240 ±0.018	1.977 ±0.011	2.046 ±0.009
$k_{-GP,SS,min}$ (s^{-1})	0.0118 ±0.0011	0.0091 ±0.0032	0.0123 ±0.0004	0.00752 ±0.00025	0.00880 ±0.00021
$k_{-GP,SS,max}$ (s^{-1})	0.0203 ±0.0014	0.019 ±0.005	0.0192 ±0.0005	0.01248 ±0.00034	0.01491 ±0.00028
$K_{-GP,SS,min}$ (M)	0.0059 ±0.0006	0.0044 ±0.0016	0.00551 ±0.00021	0.00380 ±0.00013	0.00434 ±0.00011
$K_{-GP,SS,max}$ (M)	0.0175 ±0.0028	0.020 ±0.013	0.0133 ±0.0008	0.0105 ±0.0007	0.0118 ±0.0005
$k_{GR,SS,max}$ ($M^{-1}s^{-1}$)	0.84 ±0.14	1.1 ±0.4	0.80 ±0.07	0.79 ±0.05	0.80 ±0.04
$K_{-GR,SS,min}$ (M) [a]	0.0236 ±0.0023	0.018 ±0.007	0.0240 ±0.0027	0.0159 ±0.0016	0.0194 ±0.0012
$(k_{GP,SS}/k_{GR,SS})_{min}$	1.38 ±0.32	0.9 ±0.7	1.81 ±0.19	1.51 ±0.14	1.57 ±0.10
$k_{TPR,SS,max}$ (s^{-1})	0.0085 ±0.0017	0.010 ±0.006	0.0068 ±0.0007	0.0050 ±0.0004	0.00564 ±0.00035
$(k_{TPR,SS}/k_{-GP,SS})_{max}$	0.72 ±0.20	1.1 ±0.9	0.55 ±0.07	0.66 ±0.07	0.61 ±0.05
$K_{TRP,SS,min}$ [a]	1.38 ±0.32	0.9 ±0.7	1.81 ±0.19	1.51 ±0.14	1.57 ±0.10
$(k_{TRP,SS} + k_{-GR,SS})_{min}$ (s^{-1}) [a]	0.0203 ±0.0014	0.019 ±0.005	0.0192 ±0.0005	0.01248 ±0.00034	0.01491 ±0.00028
Adj. R^2	0.999956	0.999517	0.999991	0.999995	0.99986 ±0.00012

**B**	**5 μM H_2O_2**	**10 μM H_2O_2**	**20 μM H_2O_2**	**50 μM H_2O_2**	**200 μM H_2O_2**	**Mean**
$\gamma = k_{GR,SS} + k_{GP,SS}$ ($M^{-1}s^{-1}$)	0.99 ±0.16	0.79 ±0.11	0.691 ±0.029	0.658 ±0.030	0.596 ±0.035	0.654 ±0.018
$\delta = k_{-GP,SS} + k_{TPR,SS}$ (s^{-1})	0.019 ±0.012	0.021 ±0.010	0.0122 ±0.0024	0.0072 ±0.0026	0.008 ±0.004	0.0095 ±0.0016
$\theta = k_{GP,SS} k_{-GP,SS}$ ($M^{-1}s^{-2}$)	0.011 ±0.009	0.010 ±0.007	0.0056 ±0.0012	0.0031 ±0.0009	0.0028 ±0.0011	0.0036 ±0.0006
$k_{GP,SS,min}$ ($M^{-1}s^{-1}$)	0.6 ±0.6	0.5 ±0.4	0.46 ±0.13	0.43 ±0.20	0.35 ±0.22	0.43 ±0.10
$k_{GP,SS,max}$ ($M^{-1}s^{-1}$)	0.99 ±0.16	0.79 ±0.11	0.691 ±0.029	0.658 ±0.030	0.596 ±0.035	0.654 ±0.018
$k_{-GP,SS,min}$ (s^{-1})	0.011 ±0.009	0.013 ±0.008	0.0081 ±0.0018	0.0047 ±0.0014	0.0046 ±0.0019	0.0057 ±0.0009
$k_{-GP,SS,max}$ (s^{-1})	0.019 ±0.012	0.021 ±0.010	0.0122 ±0.0024	0.0072 ±0.0026	0.008 ±0.004	0.0095 ±0.0016
$K_{-GP,SS,min}$ (M)	0.011 ±0.010	0.016 ±0.011	0.0117 ±0.0027	0.0072 ±0.0022	0.0078 ±0.0033	0.0087 ±0.0015
$K_{-GP,SS,max}$ (M)	0.03 ±0.05	0.04 ±0.05	0.027 ±0.012	0.016 ±0.013	0.023 ±0.023	0.022 ±0.008
$k_{GR,SS,max}$ ($M^{-1}s^{-1}$)	0.4 ±0.6	0.3 ±0.4	0.23 ±0.14	0.22 ±0.21	0.25 ±0.22	0.23 ±0.10
$K_{-GR,SS,min}$ (M) [a]	0.05 ±0.04	0.07 ±0.05	0.047 ±0.011	0.029 ±0.009	0.031 ±0.013	0.035 ±0.006
$(k_{GP,SS}/k_{GR,SS})_{min}$	1.4 ±2.8	1.6 ±2.7	1.9 ±1.5	1.9 ±2.4	1.4 ±1.9	1.8 ±1.0
$k_{TPR,SS,max}$ (s^{-1})	0.008 ±0.015	0.008 ±0.013	0.0042 ±0.0030	0.0024 ±0.0030	0.003 ±0.004	0.0033 ±0.0019
$(k_{TPR,SS}/k_{-GP,SS})_{max}$	0.7 ±1.8	0.6 ±1.3	0.5 ±0.4	0.5 ±0.7	0.7 ±1.1	0.5 ±0.4
$K_{TRP,SS,min}$ [a]	1.4 ±2.8	1.6 ±2.7	1.9 ±1.5	1.9 ±2.4	1.4 ±1.9	1.8 ±1.0
$(k_{TRP,SS} + k_{-GR,SS})_{min}$ (s^{-1}) [a]	0.019 ±0.012	0.021 ±0.010	0.0122 ±0.0024	0.0072 ±0.0026	0.008 ±0.004	0.0095 ±0.0016
Adj. R^2	0.997829	0.998994	0.999837	0.999736	0.99964	

[a] Assuming $k_{-GR,SS} = k_{-GP,SS}$.

$$K_{TRP,SS} = \frac{k_{TRP,SS}}{k_{TPR,SS}} > \frac{\theta}{\delta\gamma - \theta} \qquad (26)$$

$$k_{TRP,SS} + k_{-GR,SS} > \begin{cases} \delta \Leftarrow \delta\gamma \le 2\theta \\ 2\sqrt{\frac{\theta}{\gamma}\left(\delta - \frac{\theta}{\gamma}\right)} \Leftarrow \delta\gamma > 2\theta \end{cases} \qquad (27)$$

Returning to the thiol-disulfide exchange data obtained in the absence of H_2O_2 (Figure S2) and fitting equation (24) to the first 4 min of the time course of the fraction of 2DS dimeric units yields the estimates in Figure S8.

Altogether, the results from these two sets of experiments with and without H_2O_2, summarized in Table S2, show the following notable features:

1. Glutathionylation and deglutathionylation of Prdx2 disulfides by thiol-disulfide exchange occurs with rate constants ≈1.5 $M^{-1}s^{-1}$, and 0.02 s^{-1}, respectively. Thus, the first equilibrium with 8 mM GSH establishes within about 0.5 min.
2. The C_P-glutathionylated product is preferred over the C_R-glutathionylated one, both kinetically $(\frac{k_{GP,SS}}{k_{GR,SS}} > 1.6)$ and thermodynamically $(K_{TRP,SS} > 1.6)$ (Table S2). A preference for glutathionylation at one thiol *versus* the other in the same site is also consistent with the following two findings from MS analysis. First, no diglutathionylated monomers were detected in the experiment shown in Figure 4A of (Peskin et al., 2016) for glutathionylation in the absence of H_2O_2. Note that if the two active sites in a dimer were indiscriminately glutathionylated at either C_P or C_R, and GSH-mediated deglutathionylation is negligible, then diglutathionylated monomers

Table S2. Summary of best-fit estimates and bounds obtained from the data shown in Figure S9A-D. *Columns A, B reproduce the rightmost columns of Table S1A, B (respectively) for ease of comparison, columns C, D show the values obtained from the data shown in Figure S**9**C, D with the standard errors from the fits to the respective single experiments. The last column shows the variance-weighted means of the values in columns A and B.*

	**A** (n=3)	**B** (n=4)	**C** (n=1)	**D** (n=1)	**Mean(A,B)**
$\gamma = k_{GR,SS} + k_{GP,SS}$ ($M^{-1}s^{-1}$)	2.046 ±0.009	0.654 ±0.018	3.2 ±0.7	1.94 ±0.11	1.770 ±0.008
$\delta = k_{-GP,SS} + k_{TPR,SS}$($s^{-1}$)	0.01491 ±0.00028	0.0095 ±0.0016	0.123 ±0.034	0.012 ±0.005	0.01475 ±0.00027
$\theta = k_{GP,SS} k_{-GP,SS}$ ($M^{-1}s^{-2}$)	0.0177 ±0.0004	0.0036 ±0.0006	0.33 ±0.17	0.013 ±0.005	0.01306 ±0.00035
$k_{GP,SS,min}$ ($M^{-1}s^{-1}$)	1.28 ±0.04	0.43 ±0.10	2.7 ±1.6	1.1 ±0.6	1.170 ±0.035
$k_{GP,SS,max}$ ($M^{-1}s^{-1}$)	2.046 ±0.009	0.654 ±0.018	3.2 ±0.7	1.94 ±0.11	1.770 ±0.008
$k_{-GP,SS,min}$ (s^{-1})	0.00880 ±0.00021	0.0057 ±0.0009	0.10 ±0.06	0.0067 ±0.0025	0.00865 ±0.00021
$k_{-GP,SS,max}$ (s^{-1})	0.01491 ±0.00028	0.0095 ±0.0016	0.123 ±0.034	0.012 ±0.005	0.01475 ±0.00027
$K_{-GP,SS,min}$ (M)	0.00434 ±0.00011	0.0087 ±0.0015	0.032 ±0.021	0.0034 ±0.0013	0.00436 ±0.00011
$K_{-GP,SS,max}$ (M)	0.0118 ±0.0005	0.022 ±0.008	0.045 ±0.034	0.011 ±0.010	0.0118 ±0.0005
$k_{GR,SS,max}$ ($M^{-1}s^{-1}$)	0.80 ±0.04	0.23 ±0.10	0.5 ±1.7	0.9 ±0.6	0.72 ±0.04
$K_{-GR,SS,min}$ (M) [a]	0.0194 ±0.0012	0.035 ±0.006	0.13 ±0.09	0.014 ±0.005	0.0200 ±0.0012
$(k_{GP,SS}/k_{GR,SS})_{min}$	1.57 ±0.10	1.8 ±1.0	5. ±20.	1.2 ±1.2	1.58 ±0.10
$k_{TPR,SS,max}$ (s^{-1})	0.00564 ±0.00035	0.0033 ±0.0019	0.02 ±0.07	0.006 ±0.005	0.00556 ±0.00034
$(k_{TPR,SS}/k_{-GP,SS})_{max}$	0.61 ±0.05	0.5 ±0.4	0.2 ±0.7	0.8 ±1.0	0.61 ±0.05
$K_{TRP,SS,min}$[a]	1.57 ±0.10	1.8 ±1.0	5. ±20.	1.2 ±1.2	1.58 ±0.10
$(k_{TRP,SS}+k_{-GR,SS})_{min}$ (s^{-1}) [a]	0.01491 ±0.00028	0.0095 ±0.0016	0.123 ±0.034	0.012 ±0.005	0.01475 ±0.00027

[a] Assuming $k_{-GR,SS} = k_{-GP,SS}$.

should be half as abundant as monoglutathionylated ones under these conditions (see Box 1). Second, the peptide analysis in Figure S4B shows more NEM derivatization on C_R than on C_P, which supports a preference for glutathionylation at C_P. However, the significant glutathionylation at C_R already by 15 s (Figure S4B) and the quick formation of MS-detectable diglutathionylated dimers (Figure 5A) strongly suggest that the value of $k_{GR,SS}$ is close to its upper bound in Table S2. The just moderate preference for

Box 1: Relating the fraction of diglutathionylated monomers upon incubation of Prdx2-SS with GSH in the absence of H_2O_2

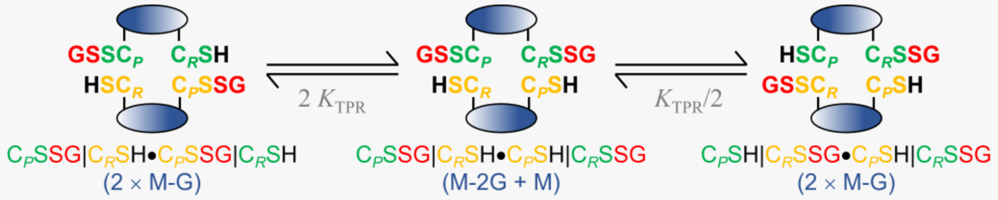

The figure above illustrates the relationship between active site and monomer glutathionylation. The S atoms from C_R and C_P belonging to the same monomer are written in the same color (green or yellow), and the glutathionyl moiety is highlighted in red. The multicolored text below each dimer shows its representation in the notation adopted throughout the text, and the blue text in parentheses below that indicates the types of monomers composing each dimer. Of note, diglutathionylated and ungltutathionylated monomers occur only when one of the active sites is glutathionylated at C_P and the other one at C_R. K_{TPR} is the per-site equilibrium constant for $C_P \rightarrow C_R$ GS transfer.

If TDE between a Prdx2 disulfide site and GSH is $1/K_{TPR}$-fold as likely to yield the C_P-glutathionylated product as the C_R-glutathionylated one, then the probability of the site being glutathionylated at C_P is $p_P = 1/(1+K_{TPR})$. If, additionally, the glutathionylation preferences of each site in a dimer are independent, then the relative frequencies of dimers with C_PC_P, C_PC_R and C_RC_R glutathionylation follow a binomial distribution: $f_{PP} = p_P^2 = 1/(1+K_{TPR})^2$, $f_{PR} = 2p_P p_R = 2K_{TPR}/(1+K_{TPR})^2$ and $f_{RR} = (1-p_P)^2 = K_{TPR}^2/(1+K_{TPR})^2$, respectively. These translate into the following relative monomer frequencies: $f_{\text{M-2G}} = f_{\text{M}} = f_{PR}/2 = K_{TPR}/(1+K_{TPR})^2$, $f_{\text{M-G}} = f_{PP} + f_{RR} = (1+K_{TPR}^2)/(1+K_{TPR})^2$. The ratio between the frequencies of diglutathionylated and monoglutathionylated monomers ($f_{\text{M-2G}}/f_{\text{M-G}} = K_{TPR}/(1+K_{TPR}^2)$, plotted below), attains a maximal value of 1/2 if there is no preference for glutathionylation at either thiol, and is lower otherwise.

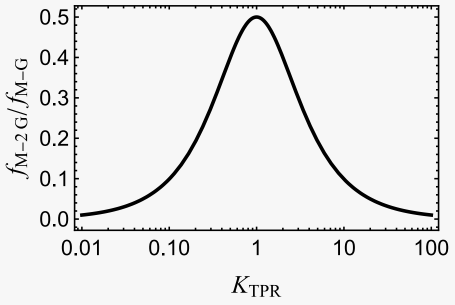

glutathionylation at C_P that this implies is not inconsistent with the results in Figure 4A of (Peskin et al., 2016). Indeed, even a moderate preference might place the fraction of diglutathionylated monomers below detection limits, because in this experiment the fraction of monoglutathionylated monomers does not exceed ~15% and the detection of ungltutathionylated monomers suggests that there is significant GSH-dependent deglutathionylation already by 5 minutes.

3. The direct transfer of the glutathionyl moiety between C_P and C_R is very slow. Equation (16) shows that the estimated value of $k_{TPR,SS}$ decreases from its already low upper limit ($k_{TPR,SS,\max} = 0.00556 \pm 0.00034\ \text{s}^{-1}$, Table S2) to 0 as $k_{GR,SS}$ approaches its upper limit consistent with the γ, δ and θ estimates. Therefore, the above-discussed evidence that $k_{GR,SS}$ is close to its upper limit also implies that $k_{TPR,SS}$ is very low. Moreover, from the microreversibility constraint (8) and from the inferences that $k_{-GR,SS} \approx k_{-GP,SS}$ and that $k_{GP,SS} / k_{GR,SS}$ can be as low as 1.6 (Table S2) it follows that $k_{TRP,SS}$ is very low as well.
4. The C_P-bound glutathionyl group is more prone to leave, reforming the disulfide, than to transfer to C_R ($\frac{k_{TPR,SS}}{k_{-GP,SS}} < 0.61$) (Table S2). In this case, the inference that $k_{GR,SS}$ approaches its upper limit implies that this ratio is much lower than 0.61.
5. The equilibrium constant for deglutathionylation from C_P is in the physiological GSH concentration range $(4.4\ \text{mM} < K_{-GP,SS} < 12.\ \text{mM})$, whereas that for deglutathionylation from C_R exceeds 18 mM (Table S2).
6. The rate constant for sulfenylation of the C_PSH|C_RSSG•C_PSSC_R thiol by H_2O_2 exceeds 750 $M^{-1}s^{-1}$. This is roughly estimated from the bound $k_{TRP,SS} + k_{-GR,SS} > 0.015\ \text{s}^{-1}$ (Table S2) and from the observation that 20 μM H_2O_2 already causes full kinetic saturation, which means that in this condition the sulfenylation outcompetes both processes blocking the peroxidatic thiol (Figure S9E&F). The 750 $M^{-1}s^{-1}$ value is a conservative underestimate of the rate constant, but it highlights that the C_PSH|C_RSSG•C_PSSC_R thiol retains a substantial reactivity despite the strong distortion of the active site expected from glutathionylation at C_R.

3.2 Thiol-disulfide exchange between GSH and the disulfide in partially glutathionylated dimers, and potential influence of glutathionylation at one site on glutathionylation of the other

3.2.1 Analysis of the experiments in the absence of H_2O_2

Upon incubation of Prdx2-SS with 4 mM GSH in the absence of H_2O_2 (Figure S2) monomers start accumulating after a few minutes, reflecting the attack of the second Prdx2 disulfide by GSH. We next extended the model to determine whether both active sites in each dimeric unit react similarly of whether glutathionylation of one active site influences the exchange reaction at the other. (Peskin et al., 2020) have previously shown that the two active sites operate cooperatively for sulfenylation, disulfide formation and reduction. The same likely happens for glutathionylation, as this modification should strongly impact Prdx2 structure compared with the interdimer disulfide. Indeed, the simple graphical representation in Figure S11 supports this expectation. Should the glutathionylation state of the first active site in a dimeric unit not influence the reactivity of the second site with GSH, as reduction by GSH progresses the fraction of dimeric units with 0, 1 and 2 disulfides would show a binomial dependence on the total fraction, f_R, of reduced sites. That is, $f_0 = f_R^2,\ f_1 = 2 f_R(1-f_R),\ f_2 = (1-f_R)^2$. Instead, the experimental results point to a deficit of 1DS dimers and a slight excess of 2DS dimers and monomers relative to this expectation, both for the experiments in the presence of catalase (Figure S11A-D) and for those in the presence of H_2O_2 where conversion to the monomer occurs much more rapidly (Figure S11E-H). This indicates a positive cooperativity: *i. e.*, glutathionylation of the first disulfide (thermodynamically and/or kinetically) facilitates glutathionylation of the second.

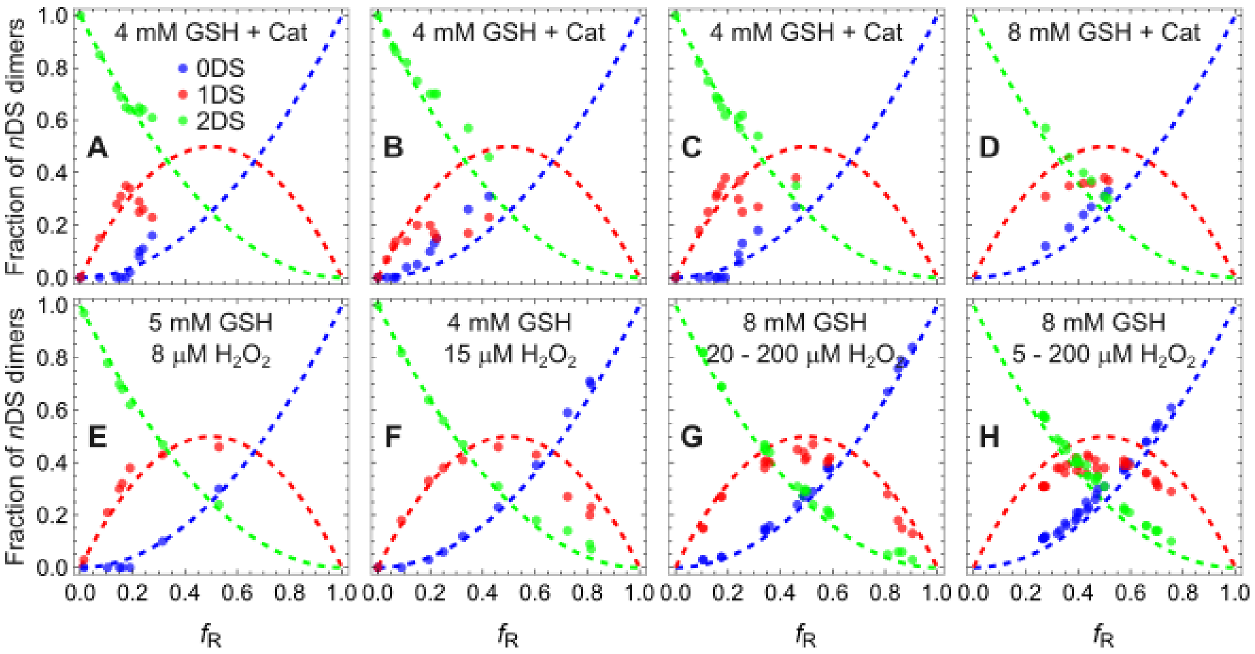

Figure S11. Fraction of dimers with 0, 1, or 2 disulfides as function of the total fraction of reduced sites in the experiments from Figures S2 and S5. *The fractions of monomers (f_0, blue), 1-disulfide dimers (f_1, red) and 2-disulfide dimers (f_2, green) at each time point were plotted as function of $f_R = f_0 + f_1/2$, The dashed lines indicate the theoretical expectation if the glutathionylation state of one active site does not influence the reactivity of the second site in the dimer. The apparent slight excess of 1DS dimers at low fractions of non-disulfide sites is likely due to a high quantification threshold for monomers in the SDS-PAGE gels.*

MS analysis shows that in the conditions of Figure S2 most of the monomeric species are glutathionylated and 1DS dimer with no GSH was not observed. In the experiments in Figure S2, the fraction of 2DS dimeric units remains almost constant for 35 min after the initial fast decrease described in the previous section, and that of monomers shows a slow increase over that long period. This shows that deglutathionylation by GSH is very slow.

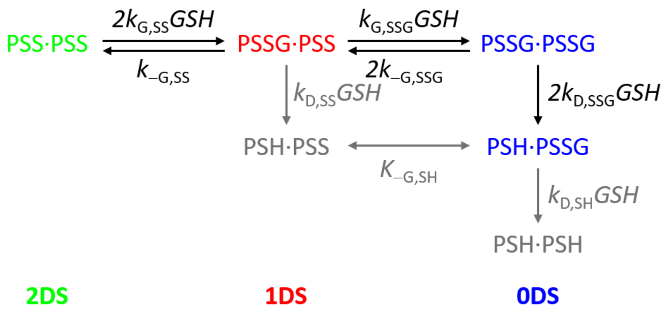

Figure S12. Reaction scheme underlying the model used to fit the time course of molecular species upon incubation of Prdx2-SS with GSH. *The symbols PSH, PSS, and PSSG denote active sites in reduced (thiol), disulfide and glutathionylated forms, respectively. The processes and species in gray were neglected in the final statistical model used to estimate the kinetic parameters.*

Fitting a suitable mechanistic model to the time courses from these experiments may allow to quantify the phenomena discussed in the previous paragraphs. However, consideration of glutathionylation products at each active site, transfer of the glutathionyl moiety between C_P and C_R, and GSH-mediated deglutathionylation of each glutathionylated product yields a quite complex reaction network and model. On the other hand, non-reducing SDS-PAGE gels do not allow to discriminate between glutathionylated and reduced sites, nor between C_P and C_R glutathionylation. Hence, whereas 2DS dimers represent a well-defined chemical species, 1DS dimers may be either glutathionylated or reduced at one active site, and 0DS dimers (running as monomers in the gels) may be either glutathionylated or reduced at each of the two active sites. Consequently, the data from SDS-PAGE experiments has a limited ability to identify the kinetic parameters of such a complex reaction network. Therefore, we considered the simplified reaction scheme in Figure S12, which does not discriminate between C_P and C_R glutathionylation. Because no unglutathionylated 1DS dimers were detected by MS and the parameter $k_{D,SH}$ is not identifiable, we neglected the processes in gray in Figure S12. We also neglected the reversal of the GSH-mediated deglutathionylation reactions, because the GSSG/GSH concentration ratio remains very low throughout the experiments at these low Prdx2-SS/GSH ratios. For this reason too, we treated all the TDE reactions as pseudo-first-order processes. Therefore, we estimated the remaining parameters based on the following system of linear ordinary differential equations (**Model 3**):

$$\frac{d\,PSS{\bullet}PSS}{dt} = k_{-\mathrm{G,SS}}PSSG{\bullet}PSS - 2k_{\mathrm{G,SS}}GSH\ PSS{\bullet}PSS$$

$$\frac{d\,PSSG{\bullet}PSS}{dt} = 2k_{\mathrm{G,SS}}GSH\ PSS{\bullet}PSS + 2k_{-\mathrm{G,SSG}}PSSG{\bullet}PSSG -$$

$$-\left(k_{-\mathrm{G,SS}} + k_{\mathrm{G,SSG}}GSH\right)PSSG{\bullet}PSS$$

$$\frac{d\,PSSG{\bullet}PSSG}{dt} = k_{\mathrm{G,SSG}}GSH\ PSSG{\bullet}PSS - 2\left(k_{-\mathrm{G,SSG}} + k_{\mathrm{D,SSG}}GSH\right)PSSG{\bullet}PSSG$$

$$\frac{d\,PSH{\bullet}PSSG}{dt} = 2k_{\mathrm{D,SSG}}GSH\ PSSG{\bullet}PSSG$$

$$PSS{\bullet}PSS(0) = (1 - f_{1,0})Prdx2_{\mathrm{Tot}}\ /\ 2$$

$$PSSG{\bullet}PSS(0) = PSSG{\bullet}PSSG(0) = PSH{\bullet}PSSG(0) = 0$$

Here, $f_{1,0}$ and $Prdx2_{\text{Tot}}$ stand, respectively, for the initial fraction of 1DS dimeric units (upper gel band) and the total concentration of Prdx2 monomer in the samples, both treated as fixed parameters. Because the closed form analytical solution of this system is very complicated, it proved computationally more effective and accurate to solve numerically. We computed the time dependent fractions of 0DS, 1DS and 2DS dimeric units as

$$f_0(t) = 2\frac{PSSG\bullet PSSG(t) + PSH\bullet PSSG(t)}{Prdx2_{\text{Tot}}},$$

$$f_1(t) = f_{1,0} + \frac{2\ PSSG\bullet PSS(t)}{Prdx2_{\text{Tot}}},$$

$$f_2(t) = 2\frac{PSS\bullet PSS(t)}{Prdx2_{\text{Tot}}},$$

using the ParametricNDSolveValue function, and fitted them to the time series using the NonlinearModelFit function in Wolfram *Mathematica*™ v.14.0.0.0 (Wolfram Research Inc., 2024).

In order to minimize correlations between the estimates for distinct parameters and obtain tighter estimates for the extent of cooperativity, the model was reparametrized by replacing the parameters at the left-hand side of the arrows by the aggregates in the right hand side: $k_{\text{G,SS}} \to k_{-\text{G,SS}} / K_{-\text{G,SS}}$, $k_{-\text{G,SSG}} \to r_{-\text{G,SSG}} k_{-\text{G,SS}}$, $k_{\text{G,SSG}} \to r_{-\text{G,SSG}} k_{-\text{G,SS}} R_{-\text{G,SSG}} K_{-\text{G,SS}}$. The five adjustable parameters were thus: $k_{-\text{G,SS}}$, $k_{\text{D,SSG}}$, $K_{-\text{G,SS}}$, $r_{-\text{G,SSG}}$ and $R_{-\text{G,SSG}}$. The last three of these parameters represent, respectively, the equilibrium constant for deglutathionylation by TDE between the GS adduct and the free thiol in the active site in dimers that carry a disulfide at the other site, the $k_{-\text{G,SSG}} / k_{-\text{G,SS}}$ ratio and the $K_{-\text{G,SSG}} / K_{-\text{G,SS}}$ ratio. In order to obtain suitable initial guesses for all the adjustable parameters, we initially set $r_{-\text{G,SSG}} = R_{-\text{G,SSG}} = k_{\text{D,SSG}} = 0$ and fitted the model to the first 60 s of the time series to estimate $k_{-\text{G,SS}}$, and $K_{-\text{G,SS}}$. Then, using these first estimates as initial guesses for the corresponding parameters we set just $k_{\text{D,SSG}} = 0$ and fitted the model to the whole time series to estimate the previous two parameters as well as $r_{-\text{G,SSG}}$ and $R_{-\text{G,SSG}}$. Finally, using the estimates for the previous four parameters as initial guesses we fitted the model with the five parameters left adjustable. We chose the estimates obtained from the statistical model, between the last two, that yielded the lowest value of the Akaike Information Criterion with small-sample correction (AICc). Estimates of the other parameters based on fits with distinct reparameterizations of the model are shown in gray in the table in Figure S13.

Although the fits do not fully capture the overshoot dynamics of $f_1(t)$, the three independent experiments yield consistent estimates for the various parameters (Figure S13). They show the following notable features:

1. For dimers with the second site in disulfide form, the fits yield a self-deglutathionylation equilibrium constant $K_{-G,SS} = 21. \pm 3.$ mM. Comparing the equilibrium solution for Model 3 with $k_{D,SSG} = 0$ to that for the corresponding model explicitly accounting for

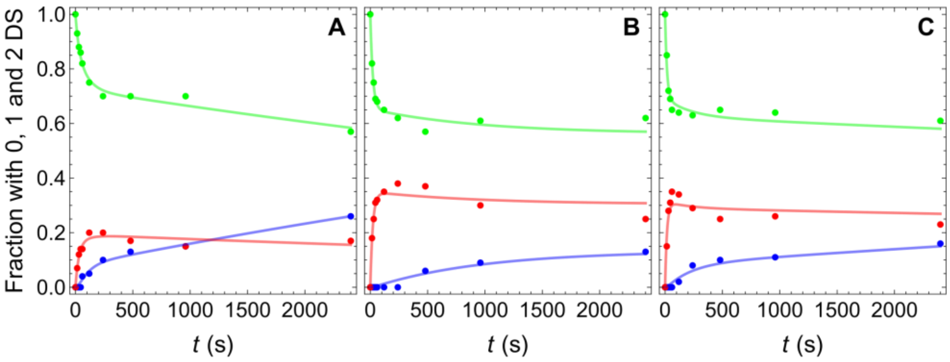

	A	B	C	Mean±SEM
$K_{-G,SS}$ (mM)	30.±1.	14.8±0.5	17.2±0.7	21. ±3.
$R_{-G,SSG}$	0.14±0.03	0.31±0.09	0.22±0.01	0.22±0.03
$k_{-G,SS}$ (s^{-1})	0.022±0.002	0.029±0.003	0.040±0.006	0.030±0.003
$r_{-G,SSG}$	0.3±0.1	0.016±0.009	0.023±0.006	0.11±0.05
$k_{D,SSG}$ ($M^{-1}s^{-1}$)	0.13±0.03	-	-	(0.13)
$k_{G,SS}$ ($M^{-1}s^{-1}$)	0.73±0.06	2.0±0.2	2.3±0.2	1.7±0.3
$K_{-G,SSG}$ (mM)	4.0±0.7	5.±1.	3.9±0.7	4.1±0.1
$k_{-G,SSG}$ (s^{-1})	(6.±3.)×10^{-3}	(5.±2.)×10^{-4}	(9.±4.)×10^{-4}	(2.±1.)×10^{-3}
$k_{G,SSG}$ ($M^{-1}s^{-1}$)	1.5±0.5	0.10±0.03	0.24±0.07	0.6±0.3
Adj. R^2	0.9991	0.997	0.997	-

Figure S13. Fits of Model 3 to the densitometry analysis of the fraction time courses for the gels shown in Figure S2. *Blue, red and green indicate the experimental (dots) or fitted (lines) fractions of dimeric units with 0, 1 or 2 disulfides, respectively. The table presents the best-fit parameters ± SEM for the parameterization described in the text (black) and for the remaining equilibrium and rate constants (gray).*

glutathionylation at C_P or C_R reveals that $K_{-G,SS} = \frac{1}{\frac{1}{K_{-GP,SS}} + \frac{1}{K_{-GR,SS}}} < \min\left(K_{-GP,SS}, K_{-GR,SS}\right)$. Thus, the results discussed in the previous section yielding $K_{-GP,SS} \leq 12.\,\text{mM} < K_{-GR,SS}$ (Table S2) indicate that $K_{-G,SS} \leq 12.\,\text{mM}$. However, there is still reasonable agreement, considering that these estimates were obtained from distinct sets of experiments. In any case, the consequence is that at GSH concentrations in the <10 mM range, Prdx2 becomes only partially glutathionylated by TDE.

2. In turn, for dimeric units with one site glutathionylated the self-deglutathionylation equilibrium constant for the other, $K_{-G,SSG} = 4.1 \pm 0.1\,\text{mM}$, is in the physiological GSH concentration range. Thus, glutathionylation at one site stabilizes the glutathionylated product at the other. This agrees with the positive cooperativity highlighted in Figure S11.
3. This thermodynamic stabilization mainly reflects a slower self-deglutathionylation when both sites are glutathionylated (half-life (log(2)/(0.002) s^{-1} = 3.×10^2 s) compared with one order of magnitude faster when the other site is a disulfide (log(2)/0.30 s^{-1} = 23. s).
4. The estimate for $k_{G,SS}$ is in excellent agreement with that for γ from Model 2 (Table S2).

5. GSH-mediated deglutathionylation is much slower than the initial glutathionylation step, and is thus the rate-limiting step in Prdx2-SS reduction by GSH. This conclusion ensues from the following considerations. The great excess of GSH over Prdx2 in these experiments makes GSH-mediated deglutathionylation essentially irreversible, causing a progressive accumulation of monomers and depletion of dimers. It is evident in Figure S13 that this process occurs only at a very slow rate, compared to the initial rapid decrease in 2DS dimers. This slowness also translated into the estimates for $k_{D,SSG}$ obtained from two of the experiments not being significantly different from 0. The presence of glutaredoxin accelerates this reaction *in vivo*, though.
6. At physiological GSH concentrations, in the absence of catalysis by glutaredoxin, GSH-mediated deglutathionylation is also much slower than deglutathionylation mediated by the free Prdx2 thiol. This conclusion follows from the initial rapid equilibration between 2DS and 1DS dimers, compared to the slow accumulation of monomers over long incubation times and is also borne out by the estimates in Figure S13.

3.2.2 Analysis of the experiments in the presence of H_2O_2

The presence of H_2O_2 in incubations of Prdx2-SS with GSH accelerates not only the decline of 2DS dimers but also the accumulation of monomer (Figure S5B and compare Figure S5C to Figure S2). To quantify and better understand the underpinnings of this phenomenon and of the observed cooperativity, we now model the evolution of the fractions of monomers, 1DS dimers and 2DS dimers under the conditions of these experiments. For that, we will first fit the model to the pertinent SDS-PAGE data to estimate the parameter values that are consistent with these data, and will then leverage on these estimates to simulate the evolution of the various species over time. To avoid the complications brought by the slow GSH-mediated Prdx2 deglutathionylation and reoxidation we focus just on the time course over the first few minutes and consider the simplified reaction scheme in the bottom part of Figure S14, justified as follows.

The previous analyses of the decay of the 2DS dimers in the same experiments indicated that at the high H_2O_2 concentrations used, the rate-limiting step in the formation of the peroxidatic sulfenic acids and then of diglutathionylated active sites is the release of the peroxidatic thiol by primary glutathionylation at C_R (dark red arrows) and/or $C_P \rightarrow C_R$ GS transfer of the C_P-glutathionylated primary product (cyan arrows). Therefore, the sulfenylation steps are not explicitly considered. In turn, the C_PSOH|C_RSSG sites should most often react with GSH to form a doubly glutathionylated site before a dimer undergoes any of the other reactions, as follows from the following two considerations. First, the 10.3 $M^{-1}s^{-1}$ rate constant for the condensation rate of the C_P-SOH in an unglutathionylated site with GSH (Figure 2C) makes the pseudo-first-order rate constant at 4 mM GSH higher than any of those listed in Table S2. Second, the evidence from the C_R mutants in Figures 2 and 6 suggests that this rate constant should be higher for a C_R-glutathionylated site.

As happens with Model 2, the $k_{GP,.}$, $k_{GR,.}$ and $k_{TPR,.}$ rate constants in the model translating the reaction scheme in Figure S14 are not identifiable from experiments at a single GSH concentration, and the latter model's lack of a tractable analytical solution prevents a rigorous calculation of the bounds implied by the fits. However, as discussed in points 2 and 3 at the end of Section 3.1, the experimental evidence allows to infer that the direct transfer of the

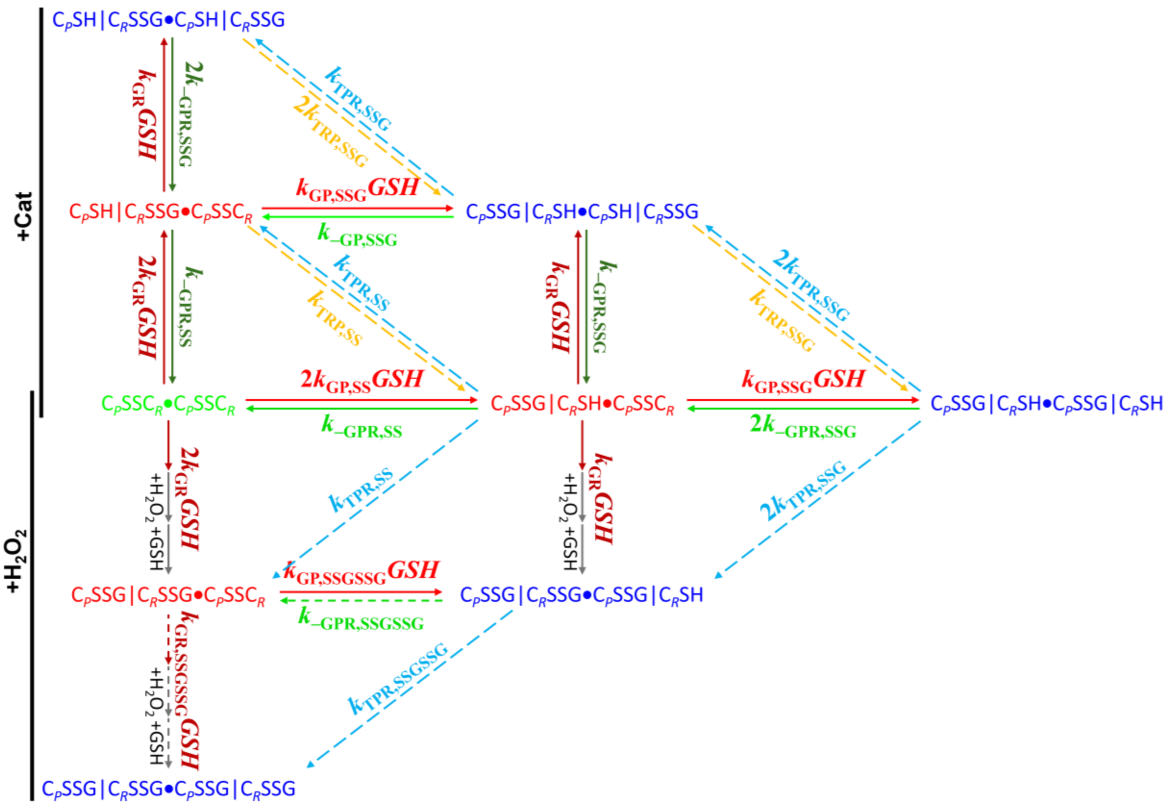

Figure S14. Reaction scheme to model the fraction time courses for the gels shown in Figures S2C, S5B&C. *Blue, red and green species symbols indicate dimeric units with 0, 1 or 2 disulfides, respectively. Reactions are colored by type: light and dark red, glutathionylation at C_P or C_R (respectively) by TDE with GSH; green, self-deglutathionylation; cyan, $C_P \rightarrow C_R$ GS transfer. In Model 4 it is assumed that in the absence of added H_2O_2 only the reactions in the upper part occur, whereas in the presence of saturating H_2O_2 only the reactions in the lower part occur due to strong competition by sulfenylation. The model neglects the processes represented by the long-dashed arrows. It also neglects the reactions represented by the short-dashed arrows because preliminary fits to the data yielded non-significant rate constant values for them.*

glutathionyl moiety between C_P and C_R is very slow. Therefore, below we neglected this transfer.

In order to constrain the $k_{-GP,.}$ estimates to be consistent with the observations from the experiments in the absence of H_2O_2 (Figure S2) we performed global fits to data from experiments including time courses for both catalase addition and high H_2O_2 concentrations (Figures S2A, S5B&C). This required considering the operation of the reaction scheme in the upper part of Figure S14 in the absence of H_2O_2. Moreover, to avoid a proliferation of nonidentifiable parameters we made the following two additional assumptions. First, that monoglutathionylation of one active site has the same effect on the properties of the other active site in a dimeric unit irrespective of the former site being glutathionylated at C_R or at C_P. Second, that the rate constants for self-deglutathionylation from C_R are identical to those for self-deglutathionylation from C_P, irrespective of the state of the other active site in the same dimeric unit (*i. e.*, we set $k_{-GPR,SS} \doteq k_{-GR,SS} = k_{-GP,SS}, k_{-GPR,SSG} \doteq k_{-GR,SSG} = k_{-GP,SSG}$). This assumption mirrors the inference $k_{-GR,SS} \approx k_{-GP,SS}$ in Section 3.1 and is supported by preliminary fits to the fraction time courses for the gels shown in Figures S2C, S5B&C, which consistently yielded estimates for $k_{-GR,SSG}$ that were not significantly different from those for

$k_{-GP,SSG}$. Because these preliminary fits also consistently yielded non-significant estimates for $k_{GR,SSGSSG}$ and $k_{-GP,SSGSSG}$, we neglected the corresponding processes.

Altogether, these reaction scheme (Figure S14) and assumptions translate into the following kinetic model (**Model 4**):

$$\frac{d\, C_PSSC_R \bullet C_PSSC_R}{dt} = k_{-GPR,SS}\ C_PSSG \,|\, C_RSH \bullet C_PSSC_R + (1-\delta_{\mathrm{H_2O_2}})k_{-GPR,SS}\ C_PSH \,|\, C_RSSG \bullet C_PSSC_R - $$
$$- 2(k_{GP,SS} + k_{GR,SS})\ GSH\ C_PSSC_R \bullet C_PSSC_R$$

$$\frac{d\, C_PSSG \,|\, C_RSH \bullet C_PSSC_R}{dt} = 2k_{GP,SS}\ GSH\ C_PSSC_R \bullet C_PSSC_R + 2k_{-GP,SSG}\ C_PSSG \,|\, C_RSH \bullet C_PSSG \,|\, C_RSH +$$
$$+ (1-\delta_{\mathrm{H_2O_2}})k_{-GPR,SSG}\ C_PSSG \,|\, C_RSH \bullet C_PSH \,|\, C_RSSG -$$
$$- \left(k_{-GP,SS} + (k_{GP,SSG} + k_{GR,SSG})\ GSH\right) C_PSSG \,|\, C_RSH \bullet C_PSSC_R$$

$$\frac{d\, C_PSSG \,|\, C_RSSG \bullet C_PSSC_R}{dt} = \delta_{\mathrm{H_2O_2}} \left(k_{-GP,SSGSSG}\ C_PSSG \,|\, C_RSSG \bullet C_PSSG \,|\, C_RSH + \right.$$
$$+ 2k_{GR,SS}\ GSH\ C_PSSC_R \bullet C_PSSC_R -$$
$$\left. -(k_{GP,SSGSSG} + k_{GR,SSGSSG})\ GSH\ C_PSSG \,|\, C_RSSG \bullet C_PSSC_R \right)$$

$$\frac{d\, C_PSSG \,|\, C_RSH \bullet C_PSSG \,|\, C_RSH}{dt} = k_{GP,SSG}\ GSH\ C_PSSG \,|\, C_RSH \bullet C_PSSC_R -$$
$$- 2k_{GP,SSG}\ GSH\ C_PSSG \,|\, C_RSH \bullet C_PSSG \,|\, C_RSH$$

$$\frac{d\, C_PSSG \,|\, C_RSSG \bullet C_PSSG \,|\, C_RSH}{dt} = \delta_{\mathrm{H_2O_2}} \left(k_{GP,SGHSSG}\ GSH\ C_PSSG \,|\, C_RSSG \bullet C_PSSC_R + \right.$$
$$+ k_{GR,SSG}\ GSH\ C_PSSG \,|\, C_RSH \bullet C_PSSC_R -$$
$$\left. -k_{-GP,SSGSSG} C_PSSG \,|\, C_RSSG \bullet C_PSSG \,|\, C_RSH \right)$$

$$\frac{d\, C_PSH \,|\, C_RSSG \bullet C_PSSC_R}{dt} = (1-\delta_{\mathrm{H_2O_2}})\left(2k_{GR,SS}\ GSH\ C_PSSC_R \bullet C_PSSC_R + \right.$$
$$+ k_{-GPR,SSG}\ C_PSSG \,|\, C_RSH \bullet C_PSSG \,|\, C_RSH +$$
$$+ 2k_{-GPR,SSG}\ C_PSH \,|\, C_RSSG \bullet C_PSH \,|\, C_RSSG -$$
$$\left. -(k_{-GPR,SS} + (k_{GP,SSG} + k_{GR,SSG})\ GSH)\ C_PSH \,|\, C_RSSG \bullet C_PSSC_R \right)$$

$$\frac{d\, C_PSSG \,|\, C_RSH \bullet C_PSH \,|\, C_RSSG}{dt} = (1-\delta_{\mathrm{H_2O_2}})\left(k_{GR,SS}\ GSH\ C_PSSG \,|\, C_RSH \bullet C_PSSC_R + \right.$$
$$+ k_{GP,SSG}\ GSH\ C_PSH \,|\, C_RSSG \bullet C_PSSC_R -$$
$$\left. -2k_{-GPR,SSG}\ C_PSSG \,|\, C_RSH \bullet C_PSSG \,|\, C_RSH \right)$$

$$\frac{d\, C_PSH \,|\, C_RSSG \bullet C_PSH \,|\, C_RSSG}{dt} = (1-\delta_{\mathrm{H_2O_2}})\left(k_{GR,SSG}\ GSH\ C_PSH \,|\, C_RSSG \bullet C_PSSC_R - \right.$$
$$\left. -2k_{-GPR,SSG}\ C_PSH \,|\, C_RSSG \bullet C_PSH \,|\, C_RSSG \right)$$

$$C_PSSC_R \bullet C_PSSC_R(0) = f_{2,0} Prdx2_{Tot} / 2$$
$$C_PSSG \,|\, C_RSH \bullet C_PSSC_R(0) = f_{1,0} Prdx2_{Tot} / 2$$
$$C_PSSG \,|\, C_RSSG \bullet C_PSSC_R(0) = 0$$
$$C_PSSG \,|\, C_RSH \bullet C_PSSG \,|\, C_RSH(0) = (1 - f_{1,0} - f_{2,0}) Prdx2_{Tot} / 2$$
$$C_PSSG \,|\, C_RSSG \bullet C_PSSG \,|\, C_RSH(0) = 0$$
$$C_PSH \,|\, C_RSSG \bullet C_PSSC_R(0) = 0$$
$$C_PSSG \,|\, C_RSH \bullet C_PSH \,|\, C_RSSG = 0$$
$$C_PSH \,|\, C_RSSG \bullet C_PSH \,|\, C_RSSG = 0$$

Here, $f_{1,0}$ and $f_{2,0}$ represent the initial fractions of 1- and 2-disulfide dimeric units, respectively, and $\delta_{\mathrm{H_2O_2}}$ takes the value of 0 if H_2O_2 is absent and 1 otherwise. It is assumed that all the initially present 1-disulfide dimers and monomers are monoglutathionylated at C_P, as the MS data did not reveal substantial hyperoxidation or initial diglutathionylation. As for Model 3, we numerically computed the time dependent fractions of 0DS, 1DS and 2DS dimeric units as:

$$f_0(t) = 2(C_PSSG \mid C_R\mathrm{SH}{\bullet}\mathrm{C}_PSSG \mid C_R\mathrm{SH}(t) + C_PSSG \mid C_RSSG{\bullet}\mathrm{C}_PSSG \mid C_R\mathrm{SH}(t) + \\ + C_PSSG \mid C_RSH{\bullet}C_PSH \mid C_RSSG(t) + C_PSH \mid C_RSSG \bullet C_PSH \mid C_RSSG(t)) / Prdx2_{\mathrm{Tot}},$$

$$f_1(t) = f_{1,0} + 2\frac{C_PSSG \mid C_R\mathrm{SH}{\bullet}\mathrm{C}_PSSC_R(t) + C_PSSG \mid C_RSSG{\bullet}C_PSSC_R(t) + C_PSH \mid C_RSSG \bullet C_PSSC_R(t)}{Prdx2_{\mathrm{Tot}}},$$

$$f_2(t) = 2\frac{\mathrm{C}_PSSC_R{\bullet}\mathrm{C}_PSSC_R(t)}{Prdx2_{\mathrm{Tot}}},$$

using the ParametricNDSolveValue function in Wolfram *Mathematica*™ v.14.0.0.0 (Wolfram Research Inc., 2024).

Global fits of Model 4 to data from the experiments in Figures S2A and S5B&C still consistently yielded estimates for $k_{GR,SSG}$ that were not significantly different from those for $k_{GR,SS}$. Therefore, we set $k_{GR} \doteq k_{GR,SSG} = k_{GR,SS}$. The fits of Model 4A with the reduced set of parameters ($k_{GP,SS}$, $k_{-GPR,SS}$, k_{GR}, $k_{GP,SSG}$, $k_{-GPR,SSG}$, $k_{GP,SSGSSG}$) consistently yielded lower or not significantly different corrected Akaike Information Criterion statistics than the original model, supporting the pertinence of these simplifications. We have also fitted suitable reparametrized models to estimate relevant equilibrium constants and ratios. Namely, $K_{-GP,SS}$, $K_{-GP,SSG}$, $R_{-GP,SSG} \doteq K_{-GP,SSG} / K_{-GP,SS}$, $r_{GP,SSG} \doteq k_{GP,SSG} / k_{GP,SS}$, $r_{GP,SSGSSG} \doteq k_{GP,SSGSSG} / k_{GP,SS}$, $r_{-GPR,SSG} \doteq k_{-GPR,SSG} / k_{-GPR,SS}$ and $k_{GP,SS} / k_{GR}$. Altogether, the results, collected in the table in Figure S15, show the following notable features:

1. The estimates for $k_{GP,SS}$ from Model 4 are in good agreement with those for $k_{GP,SS,\min}$ in Table S2. However, those for $k_{-GPR,SS}$ and $K_{-GP,SS}$ are somewhat higher than those for $k_{-GP,SS,\max}$ and $K_{-GP,SS,\max}$ (respectively) in Table S2, and those for k_{GR} are lower than those for $k_{GR,SS,\max}$ in Table S2. Thus, the former fits also yield a higher value of $(k_{GP,SS} / k_{GR,SS})_{\min} = 3.21 \pm 0.23$. We consider the estimates in Table S2 more reliable, because they were derived from excellent fits of a model that relies on weaker assumptions and fewer approximations.
2. The modest positive cooperativity highlighted in Figure S11E-H appears to be mainly due to a modest positive cooperativity in the deglutathionylation rate: $r_{-GPR,SSG} = 0.55 \pm 0.05$, translating that deglutathionylation of the second active site in a dimeric unit becomes twice faster after deglutathionylation of the first site, whereas $r_{GP,SSG} = 0.99 \pm 0.08$ is not significantly different from 1. The analysis also did not reveal significant differences between the estimates for $k_{GR,SSG}$ and $k_{GR,SS}$, and equating these parameters did not

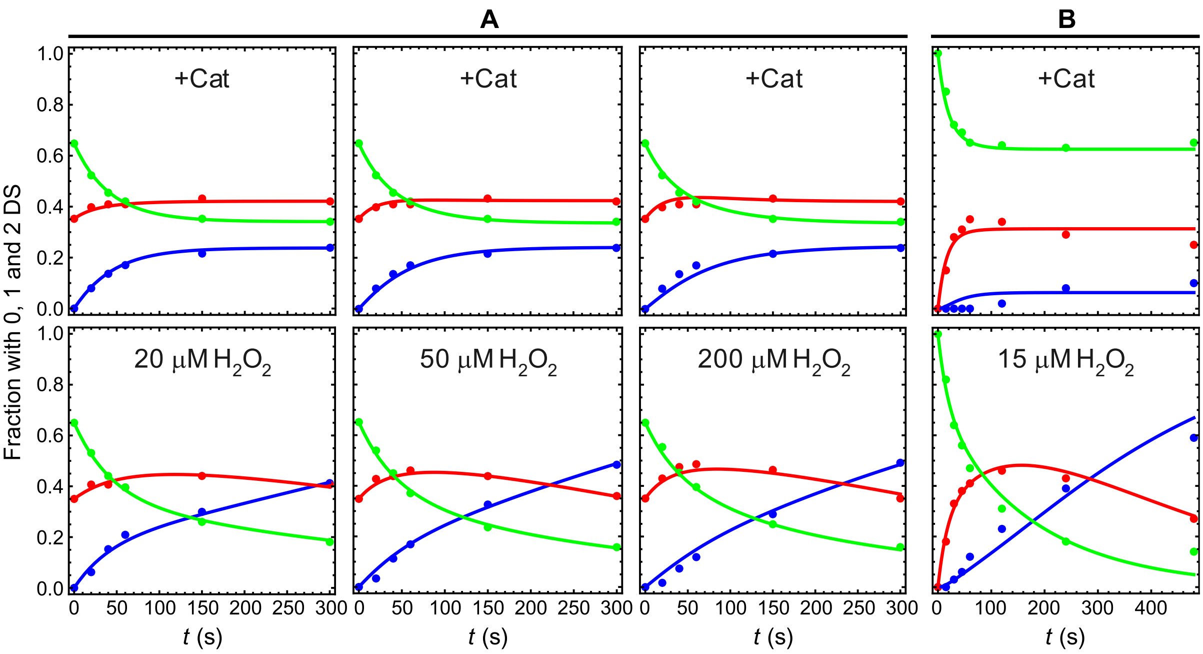

	20 μM H_2O_2	50 μM H_2O_2	200 μM H_2O_2	Mean(expmt.)±SEM[a]	15 μM H_2O_2	Mean±SEM[b]
$k_{GP,SS}$ ($M^{-1}s^{-1}$)	1.07 ±0.08	1.00 ±0.08	0.96 ±0.14	1.02 ±0.05	1.63 ±0.27	1.05 ±0.05
$k_{-GPR,SS}$ (s^{-1})	0.0168 ±0.0015	0.0165 ±0.0014	0.0162 ±0.0024	0.0166 ±0.0009	0.039 ±0.005	0.0172 ±0.0009
k_{GR} ($M^{-1}s^{-1}$)	0.230 ±0.017	0.296 ±0.017	0.310 ±0.031	0.268 ±0.011	0.82 ±0.06	0.286 ±0.011
$k_{GP,SSG}$ ($M^{-1}s^{-1}$)	1.58 ±0.13	1.03 ±0.08	0.67 ±0.11	1.03 ±0.06	1.0 ±1.3	1.03 ±0.06
$k_{-GPR,SSG}$ (s^{-1})	0.0128 ±0.0012	0.0094 ±0.0008	0.0067 ±0.0011	0.0093 ±0.0006	0.018 ±0.016	0.0093 ±0.0006
$k_{GP,SSGSSG}$ ($M^{-1}s^{-1}$)	0.44 ±0.04	0.57 ±0.04	0.52 ±0.06	0.507 ±0.026	0.89 ±0.07	0.554 ±0.025
$K_{-GP,SS}$ (mM)	15.8 ±0.5	16.7 ±0.5	16.7 ±0.9	16.30 ±0.34	24.0 ±1.9	16.54 ±0.33
$K_{-GP,SSG}$ (mM)	8.07 ±0.31	9.1 ±0.4	10.0 ±0.9	8.61 ±0.23	18. ±11.	8.61 ±0.23
$R_{-GP,SSG}$	0.76 ±0.10	0.54 ±0.07	0.40 ±0.09	0.55 ±0.05	0.5 ±0.4	0.55 ±0.05
$r_{GP,SSG}$	1.47 ±0.17	0.99 ±0.11	0.68 ±0.14	1.00 ±0.08	0.6 ±0.8	0.99 ±0.08
$r_{GP,SSGSSG}$	0.41 ±0.05	0.53 ±0.05	0.53 ±0.10	0.473 ±0.034	0.55 ±0.10	0.481 ±0.032
$r_{-GPR,SSG}$	0.71 ±0.10	0.54 ±0.07	0.42 ±0.09	0.55 ±0.05	0.5 ±0.4	0.55 ±0.05
$k_{GP,SS}/k_{GR}$	4.6 ±0.5	3.4 ±0.4	3.4 ±0.7	3.72 ±0.27	2.0 ±0.4	3.21 ±0.23
Adj. R^2	0.998986	0.999098	0.997331	–	0.993595	–

[a] Variance-weighted mean of the runs to the left
[b] Variance-weighted mean of the two experiments

Figure S15. Fits of Model 4B to the densitometry analysis of the fraction time courses for the gels shown in Figure S5B (A) as well as in Figures S2C and S5C (B) for glutathionylation of 5 µM oxidized Prdx2 treated with GSH and Cat or H_2O_2. *Blue, red and green indicate the experimental (dots) or fitted (lines) fractions of dimers with 0, 1 or 2 disulfides, respectively. The table presents the best-fit parameters ± SEM for the parameterization described in the text (black) and for the remaining equilibrium constants, rate constants and ratios thereof (gray). Fits were done in Mathematica™ v14.0.0.0 using the function NonlinearModelFit with default options.*

significantly worsen the fits. However, diglutathionylation at one active site appears to slow-down glutathionylation at C_P at the other active site in the same dimeric unit.

3. The estimates for $R_{-GP,SSG}$ are significantly different from that for $R_{-G,SSG}$ in Figure S13. This is not unexpected, as these parameters describe different phenomena. Thus, $R_{-G,SSG}$ is the extent to which self-deglutathionylation from C_P and C_R on aggregate at one active site is influenced by monoglutathionylation at the other site in the same dimeric unit. In turn, $R_{-GP,SSG}$ is the extent to which self-deglutathionylation from C_P at one site is influenced by mono- and di-glutathionylation (on aggregate) at the other site. But considering the

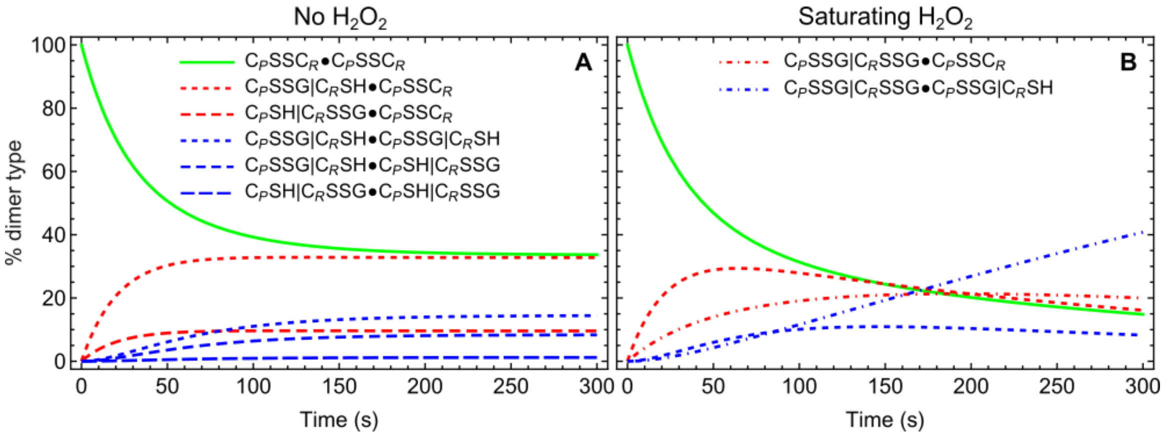

Figure S16. Simulations of the time courses of the Prdx2 species for an incubation of Prdx2 with 8 mM GSH in the absence of H_2O_2 (A) or in the presence of saturating H_2O_2 (B), based on Model 4 and the parameter estimates shown in Figure S15. *Green, 2DS dimers; red, 1DS dimers; blue, monomers in non-reducing SDS-PAGE gels.*

approximations involved in these coarse models, not too much should be read from the difference between the estimates for these two parameters.

Forward simulations of Model 4 based on the parameter estimates in Figure S15 show the time course of the various species and help understand the effects of H_2O_2 on the dynamics of dimers and monomers (Figure S16). In the absence of H_2O_2 (Figure S16A), 2DS dimers, dimers monoglutathionylated at one active site and dimeric units monoglutathionylated at both active sites (running as monomers in gels) equilibrate within 2 minutes. [As expected, this is in accordance to the experimental observations for this condition (Figures S8A-C and S15A&B top panels), and is presented here to facilitate comparison to the next simulations based on the same model.] In the presence of saturating H_2O_2 (Figure S16B), the dynamics over the first minute is mostly driven by this equilibration. However, a slow transfer of the glutathionyl moiety from C_P to C_R or direct formation of the C_R-glutathionylated product, both followed by sulfenylation and condensation with GSH, displace that equilibrium towards the formation of monomers, which accumulate over several minutes. According to the estimates in Figure S15, 21% of the C_PSSC_R•C_PSSC_R dimers and 8.3% of the $C_PSSG|C_RSH$•C_PSSC_R dimers proceed to (further) sulfenylation and diglutathionylation, after direct glutathionylation of C_R. Diglutathionylation of a site irreversibly prevents its self-deglutathionylation restoring the disulfide. The suppression of the latter reaction results in an apparent acceleration of the consumption of 2DS dimers and of monomer formation in the presence of H_2O_2.

4 References

Ngamchuea, K., Batchelor-McAuley, C., & Compton, R. G. (2016). The Copper(II)-Catalyzed Oxidation of Glutathione. *Chemistry – A European Journal*, *22*(44), 15937–15944. doi: 10.1002/chem.201603366

Peskin, A. V., Meotti, F. C., de Souza, L. F., Anderson, R. F., Winterbourn, C. C., & Salvador, A. (2020). Intra-dimer cooperativity between the active site cysteines during the oxidation of peroxiredoxin 2. *Free Radical Biology and Medicine*, *158*, 115–125. doi: 10.1016/j.freeradbiomed.2020.07.007

Peskin, A. V., Meotti, F. C., Kean, K. M., Göbl, C., Peixoto, A. S., Pace, P. E., Horne, C. R., Heath, S. G., Crowther, J. M., Dobson, R. C. J., Karplus, P. A., & Winterbourn, C. C. (2021). Modifying the resolving cysteine affects the structure and hydrogen peroxide reactivity of peroxiredoxin 2. *Journal of Biological Chemistry*, *296*, 100494. doi: 10.1016/j.jbc.2021.100494

Peskin, A. V, Pace, P. E., Behring, J. B., Paton, L. N., Soethoudt, M., Bachschmid, M. M., & Winterbourn, C. C. (2016). Glutathionylation of the Active Site Cysteines of Peroxiredoxin 2 and Recycling by Glutaredoxin. *The Journal of Biological Chemistry*, *291*(6), 3053–3062. doi: 10.1074/jbc.M115.692798

Reisz, J. A., Bechtold, E., King, S. B., Poole, L. B., & Furdui, C. M. (2013). Thiol-blocking electrophiles interfere with labeling and detection of protein sulfenic acids. *FEBS Journal*, *280*(23), 6150–6161. doi: 10.1111/febs.12535

Winterbourn, C. C., & Metodiewa, D. (1999). Reactivity of biologically important thiol compounds with superoxide and hydrogen peroxide. *Free Radical Biology and Medicine*, *27*(3–4), 322–328.

Wolfram Research Inc. (2024). *Mathematica* (Version 14). Champaign, IL, U.S.A.: Wolfram Research, Inc.
